# Clinical Classification of Cancer Cachexia: Phenotypic Correlates in Human Skeletal Muscle

**DOI:** 10.1371/journal.pone.0083618

**Published:** 2014-01-03

**Authors:** Neil Johns, Shinji Hatakeyama, Nathan A. Stephens, Martin Degen, Simone Degen, Wilfried Frieauff, Christian Lambert, James A. Ross, Ronenn Roubenoff, David J. Glass, Carsten Jacobi, Kenneth C. H. Fearon

**Affiliations:** 1 Department of Clinical and Surgical Sciences, University of Edinburgh, Edinburgh, United Kingdom; 2 Novartis Institutes for BioMedical Research Basel, Novartis Pharma AG, Basel, Switzerland; 3 Novartis Institutes for Biomedical Research, Cambridge, Massachusetts, United States of America; McGill University, Canada

## Abstract

**Background:**

Cachexia affects the majority of patients with advanced cancer and is associated with a reduction in treatment tolerance, response to therapy, and duration of survival. One impediment towards the effective treatment of cachexia is a validated classification system.

**Methods:**

41 patients with resectable upper gastrointestinal (GI) or pancreatic cancer underwent characterisation for cachexia based on weight-loss (WL) and/or low muscularity (LM). Four diagnostic criteria were used >5%WL, >10%WL, LM, and LM+>2%WL. All patients underwent biopsy of the rectus muscle. Analysis included immunohistochemistry for fibre size and type, protein and nucleic acid concentration, Western blots for markers of autophagy, SMAD signalling, and inflammation.

**Findings:**

Compared with non-cachectic cancer patients, patients with LM or LM+>2%WL, mean muscle fibre diameter was reduced by about 25% (p = 0.02 and p = 0.001 respectively). No significant difference in fibre diameter was observed if patients had WL alone. Regardless of classification, there was no difference in fibre number or proportion of fibre type across all myosin heavy chain isoforms. Mean muscle protein content was reduced and the ratio of RNA/DNA decreased in patients with either >5%WL or LM+>2%WL. Compared with non-cachectic patients, SMAD3 protein levels were increased in patients with >5%WL (p = 0.022) and with >10%WL, beclin (p = 0.05) and ATG5 (p = 0.01) protein levels were increased. There were no differences in phospho-NFkB or phospho-STAT3 levels across any of the groups.

**Conclusion:**

Muscle fibre size, biochemical composition and pathway phenotype can vary according to whether the diagnostic criteria for cachexia are based on weight loss alone, a measure of low muscularity alone or a combination of the two. For intervention trials where the primary end-point is a change in muscle mass or function, use of combined diagnostic criteria may allow identification of a more homogeneous patient cohort, reduce the sample size required and enhance the time scale within which trials can be conducted.

## Introduction

Cancer cachexia has been defined recently as a multifactorial syndrome characterised by an ongoing loss of skeletal muscle mass (with or without loss of fat mass) that cannot be fully reversed by conventional nutritional support and leads to progressive functional impairment [1]. Cachexia affects the majority of patients with advanced cancer and is associated with a reduction in treatment tolerance, response to therapy, quality of life and duration of survival. Skeletal muscle loss appears to be the most significant event in cancer cachexia and is associated with a poor outcome [1], [2]. The international consensus on the classification of cancer cachexia suggested that diagnostic criteria should take into account not only that weight loss is a signal event of the cachectic process but that the initial reserve of the patient should also be considered (either low BMI or low level of muscularity). Although the latter concept has some validation in terms of clinical risk [2], there has been no evaluation of the biological correlates in terms of changes within skeletal muscle itself.

Human skeletal muscle is composed of muscle fibres that are classified depending on their speed of contraction and predominant type of energy metabolism. Muscle fibres can be classified as type I (slow-twitch) and type II (fast-twitch) fibres based on their predominant myosin heavy chain (MyHC) isoform content. Generally, type I and type IIa fibres utilise oxidative phosphorylation, whereas type IIx and IIb fibres harness primarily anaerobic metabolism to generate ATP. Both the percentage and structural morphology of the fibre type will determine the phenotypic capacity and functional performance of any given muscle. Environmental factors in both health and disease have a direct impact leading to changes in fibre type/morphology and consequent functionality; such processes include aging, exercise, chronic disease, and cachexia [3]–[7]. The change, preservation or loss of fibres may influence clinical symptoms and there is some evidence that all types of MyHC is targeted selectively in cancer cachexia [8]. Ongoing loss of protein in muscle tissue may lead to muscle fibre shrinkage and a reduction in cross-sectional area (CSA). Equally, loss of muscle fibre CSA may lead to loss of aerobic capacity (VO_2_ max) in healthy subjects as well as cancer patients [5], [9].

Although systemic inflammation is generally thought to be an important upstream mediator of cancer cachexia [10], the precise molecular mechanisms that mediate the changes in protein synthesis and degradation that ultimately lead to atrophy of muscle fibres in cancer cachexia in humans are not known. For each animal model that has been studied, different pathways have been implicated. From such animal models there is a predominant impression that increased degradation via activation of the ubiquitin proteasome pathway (UPP) is important [10]. In contrast, human data is very limited. Activation of protein degradation via the UPP has not been a consistent finding [11] [12]. This has led to suggestions that autophagy may be important or that pathways that may influence both synthesis and degradation may be important (e.g. TGF-β/SMAD signalling) [13].

In the present study we chose to evaluate the relationship between the different cachexia definitions, systemic inflammation (serum C-reactive protein) and potential inflammatory signalling pathways within muscle (phospho-STAT3 and phospho-NFkB). We also examined for potential associations between the various cachexia definitions and activation of autophagy pathways or TGF-β/SMAD signalling.

The aim of this study was to investigate the changes in muscle fibre biology with regards to morphological structure and composition, to study alteration in various pathways that may account for altered fibre size and relate these changes to the different diagnostic criteria that have been proposed as part of the recent international consensus on the classification of cancer cachexia [1].

## Materials and Methods

### Patient Recruitment, Identification, Consent and Ethics

Patients with resectable disease and suitable for the study were identified via the upper gastrointestinal cancer multi-disciplinary team (MDT) meetings at the Royal Infirmary, Edinburgh, UK. Written consent was given prior to entry into the study. All procedures were approved by the NHS Lothian local research ethics committee. The study conformed to the standards set by the Declaration of Helsinki.

### Calculation of Weight Loss

Pre-morbid weight was recalled by the patient and verified where possible from the medical notes. Although there may be recall bias, evidence to support the reliability of self-reported weight and weight history [14], [15] is well documented. Individual weight loss was calculated and expressed as percentage of pre-morbid body weight lost.

### Classification of Cancer Cachexia

- Weight loss >5% over past 6 months (in absence of simple starvation) (WL>5%)
- Weight loss >10% over past 6 months (in absence of simple starvation) (WL>10%)
- Stature adjusted skeletal muscle index consistent with low muscularity (LM) (see ‘CT-image analysis’ for cut-offs)
- Stature adjusted skeletal muscle index consistent with low muscularity and any degree of weight loss >2% (LM + >2%WL)

### Rectus Abdominis Muscle Biopsy and Storage For Biochemical Analysis

All biopsies were taken at the start of open abdominal surgery under general anaesthesia. Patients had fasted overnight prior to surgery. The edge of the rectus abdominis was exposed and a 1 cm^3^ specimen removed using sharp dissection. The biopsy was cleaned of gross blood contamination. Obvious fat/fibrous tissue was removed prior to placement in a cryotube and being snap frozen in liquid nitrogen and stored at −80°C.

### Rectus Abdominis Muscle Sample Preparation for Cryo-Section

A 0.1–0.5 cm^3^ section of muscle was cut. Liquid nitrogen was used to cool isopentane solvent in a tube to a temperature of ∼−190°C. The section of muscle was stitched onto a segment of cork. OCT solution was placed at the junction between the cork base and the muscle. This was then lowered with the cork uppermost (i.e. muscle first) into cooled solvent and held for approximately 5 minutes (until the muscle was frozen). Samples were then stored at −80°C until use.

### CT Image Analysis

CT scans used for the analysis were done solely for routine cancer care. A transverse CT image from the third lumbar vertebrae (L3) was assessed for each scan date and tissue volumes estimated [16]. All CT images were analysed by a single trained observer. Cross-sectional area for muscle and adipose tissue was normalized for stature (cm^2^/m^2^).

Estimates of whole body stores were generated from the raw data (cm^2^) using the regression equations by Mourtzakis et al. [17], which show a close correlation between muscle and fat areas in CT images at the third lumbar vertebrae and whole body compartments of fat-free mass (FFM) and fat mass (FM) respectively.
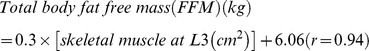


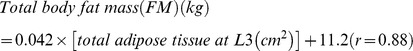
The respective indexes for FFM and FM (kg/m^2^) were calculated.

Cutoffs for low muscularity were based on a CT-based sarcopenic obesity study of cancer patients by Prado et al. (i.e., L3 skeletal muscle index: ≤38.5 cm^2^/m^2^ for women and ≤52.4 cm^2^/m^2^ for men) [18].

CT scans used were routine diagnostic staging CT scans which were performed within 30 days of a diagnosis of cancer and all were in treatment naive patients. The median time to biopsy after the CT scan was 18 days.

### Immunohistochemistry

The frozen muscle sections were co-stained for laminin (L9393, Sigma-Aldrich, Buchs, Switzerland) and myosin heavy chain type I or IIa to distinguish each fibre type (BA-D5 for type I, SC-71 for type IIa). The paraffin sections were stained for phospho-STAT3 (D3A7, Cell Signaling Technologies, Danvers, MA, USA) with a Ventana discovery XT (Roche group, Tucson, USA). Images of the entire tissue section were acquired using a VS120 slide scanner (Olympus Corporation, Tokyo, Japan). The distribution of myosin heavy chain fibre types, the cross section area of the individual fibres in the section, and the phospho-STAT3 positive nuclei and staining density were analysed using the proprietary image analysis platform ASTORIA (Automated Stored Image Analysis) developed by Novartis/Preclinical Safety.

### Tissue Preparation for DNA, RNA and Protein Extractions

Skeletal muscle tissue was minced and ground on dry ice. Aliquots were weighed using an analytical balance (Mettler Toledo) and stored at −80°C until use.

### DNA and RNA Extraction and Linearity of the Extraction Method

DNA and RNA from human skeletal muscle tissue was extracted and purified with the automated Maxwell 16 system (Promega, Duebendorf, Switzerland). To determine the linearity of the extraction methods using the Maxwell 16 system, DNA and RNA was extracted from 4 mg, 6 mg, 8 mg, and 10 mg of muscle, respectively. Calculating the total DNA and RNA content per wet weight (which in a linear extraction system should be equal for all aliquots), allowed us to define the linear range of the Maxwell 16 extraction system. Based on these preliminary studies, aliquots of 4–8 mg human skeletal muscle tissue were used for all subsequent DNA and RNA extractions. Using more starting material drastically reduced the total DNA and RNA content per wet weight (data not shown).

For DNA extraction, the Maxwell 16 LEV Blood DNA Kit (Promega) was used with a slightly adapted protocol compared with the manual's instructions. Briefly, 300 µl of Tail Lysis Buffer from the kit ReliaPrep gDNA Tissue Miniprep System (Promega) was added to minced and ground human skeletal muscle tissue in Precellys 24 lysing kit tubes. Tissue was further homogenized using the high-throughput homogenizer Precellys 24, for 10 s. After cooling on ice for 5 minutes, 30 µl of the protein K and 5 µl of the 1-Thiolglycerol solution were added. This mixture was incubated at 56°C for 2 hrs. Afterwards, the lysate was transferred into well 1 of the LEV Blood DNA cartridge, and diluted with 300 µl nuclease-free water. For the elution, 50 µl of elution buffer was added into elution tubes. The Maxwell 16 instrument was started using the DNA Blood program.

For RNA extraction, the Maxwell 16 LEV simplyRNA Tissue Kit was used (Promega), following the manual's instructions. Briefly, minced and ground human muscle tissue was incubated in 200 µl of chilled 1-Thioglycerol/Homogenization solution and further homogenized using the Precellys 24 system (see DNA). Afterwards, the samples were heated at 70°C for 2 min, then the lysates were allowed to cool down. 200 µl of lysis buffer was added to the cooled-down homogenate, mixed vigorously, followed by transfer of the total 400 µl into well 1 of the Maxwell 16 LEV cartridge. 5 µl of DNAse was added to well 4 of the cartridge and, 50 µl RNAse-free water was added to 0.5 ml Elution Tubes and the RNA extraction program was started at the Maxwell 16 instrument.

Extracted DNA and RNA were measured spectrometrically using a Trinean DropSense Instrument (Trinean, Gentbrugge, Belgium) for quantity and quality.

### Protein Extractions

To extract proteins, 300 µl of PhosphoSafe Extraction Reagent (Millipore) was added to a specific amount (between 8 and 18 mg) of homogenized human skeletal muscle tissue. To further homogenize the samples, the Precellys 24 system was used (see section above). After incubation on ice for 5 min, the lysates were spun at 800×g for 5 min at 4°C. Supernatants were transferred into new tubes and spun for another 12 min at 1600×g at 4°C. Supernatants were collected and protein concentrations measured using the BCA Protein Assay Kit (Pierce) with BSA as a standard. Afterwards, phosphatase inhibitor cocktail (Roche) was added and the samples were stored at −80°C until further use.

### Western Blots

20 µg of human skeletal muscle protein extracts (see above) in reducing Laemmli SDS sample buffer were boiled for 5 min at 95°C and then separated by SDS-PAGE on 4–20% gradient gels (Bio-Rad, Cressier, Switzerland), blotted to Nitrocellulose membranes (Bio-Rad) using the Trans-Blot Turbo Transfer System (Bio-Rad), blocked for 1 h in 5% non-fat milk in Tris-buffered saline+0.05% Tween-20, incubated overnight with primary antibody, rinsed, and incubated for 1 h with peroxidase-conjugated goat anti-rabbit IgG (1∶5000) (Santa Cruz, Heidelberg, Germany) at room temperature. Blots were developed using ECL (Roche, Rotkreuz, Switzerland) or SuperSignal West Femto substrate (Thermo Scientific, Wohlen, Switzerland) and exposed to Kodak film (Kodak, Rochester, NY, USA).

Rabbit monoclonal antibodies used were: Beclin-1 (clone D40C5), Atg5 (clone D1G9) , Atg7 (clone D12B11), Atg12 (clone D88H11), SMAD3 (clone C67H9), phospho-NF*κ*B p65 (Ser536) (clone 93H1) and α-tubulin (clone 11H10) (all from Cell Signaling Technologies, Danvers, MA, USA), phospho-SMAD3 (Ser423/Ser425, clone EP823Y) (Millipore, Billerica, MA, USA). Rabbit polyclonal antibodies used were: Gelsolin (Cell Signaling Technologies).

Western blots were analyzed densitometrically using ImageJ software version 1.45 (NIH, Bethesda, MD, USA; http://rsbweb.nih.gov/ij). Band intensity of each sample was normalised to that of α-tubulin.

### C - Reactive Protein (CRP)

Serum CRP concentration was measured with an automated immunoturbidimetric assay by clinical chemistry department, Royal infirmary Edinburgh, using blood collected from patients at the time of recruitment and before any therapeutic intervention.

### Statistical Analysis

Results are expressed as mean (± SEM). Comparisons between groups were performed using unpaired Student's t tests, whereas possible relationships were evaluated using Pearson's correlations. Results were considered significant if p values were less than 0.05. The program SPSS (version 20, SPSS, Chicago, IL, USA) was used for all the statistical tests.

## Results

### Patient Demographics

A total of 41 cancer patients with resectable UGI or pancreatic cancer were recruited. In general, patients were over 65 years of age, predominantly male and had sustained, on average, 5% loss of weight compared with pre-illness levels (Table 1). Patients were grouped based upon the concepts of the International Classification Framework [1] according to weight loss or weight loss in association with low muscularity. The specific phenotypes considered were weight loss >5% (WL>5%), weight loss >10% (WL>10%), low muscularity (LM), and LM with weight loss >2% (LM+>2% WL). Although BMI was reduced in all groups classified as cachectic, only the LM and LM+>2% WL groups had a significantly lower fat free mass index (Table 1).

**Table 1:** Demographic data of the patients involved in the study.

	All Patients (n = 41)	Weight Stable (n = 23)	Weight Loss >5% (n = 18)	p	Weight Stable (n = 30)	Weight Loss >10% (n = 11)	p	Normal Muscularity (n = 9)	Low Muscularity (n = 32)	p	Not Low Muscularity + >2% W/L (n = 24)	Low Muscularity + >2% W/L (n = 17)	p
Age	65±2	67±2	63±2	0.216	65±2	66±2	0.849	61±3	66±2	0.128	65±2	66±2	0.727
Sex (M∶F)	30∶11	19∶4	11∶7		23∶7	7∶4		4∶5	26∶6		19∶5	11∶6	
Pre Illness Weight (kg)	83±3	83±4	81±4	0.748	82±3	83±5	0.912	86±7	81±3	0.487	84±4	81±4	0.602
Weight Loss (%)	5±1	0±1	12±1	**0.000** *	2±1	15±2	**0.000** *	5±3	6±1	0.752	2±1	11±1	**0.000** *
BMI	26±1	28±1	24±1	**0.016** *	27±1	24±1	**0.048** *	29±2	25±1	**0.050** *	28±1	24±1	**0.013** *
Body Fat (kg)	17±1	18±2	16±1	0.416	17±1	16±1	0.609	16±3	17±1	0.768	18±2	16±1	0.258
Fat Free Mass Index (FFMI) (kg/m^2^)	16±0	16±1	15±1	0.057	16±1	15±1	0.143	17±1	15±0	**0.026** *	17±1	14±0	**0.001** *
CRP (mg/L)	14.5±5	16±8	12±5	0.667	13±6	18±8	0.670	15±9	14±6	0.966	10±4	20±10	0.301

Data (except gender split) are presented as mean (SEM).

= cachexia group significantly different from the non-cachexia group, (p<0.05 by Student's t test).

Abbreviations – CRP = C - reactive protein, BMI = Body Mass Index.

### Muscle Fibre Size, Number, and Type

**Figure 1:**
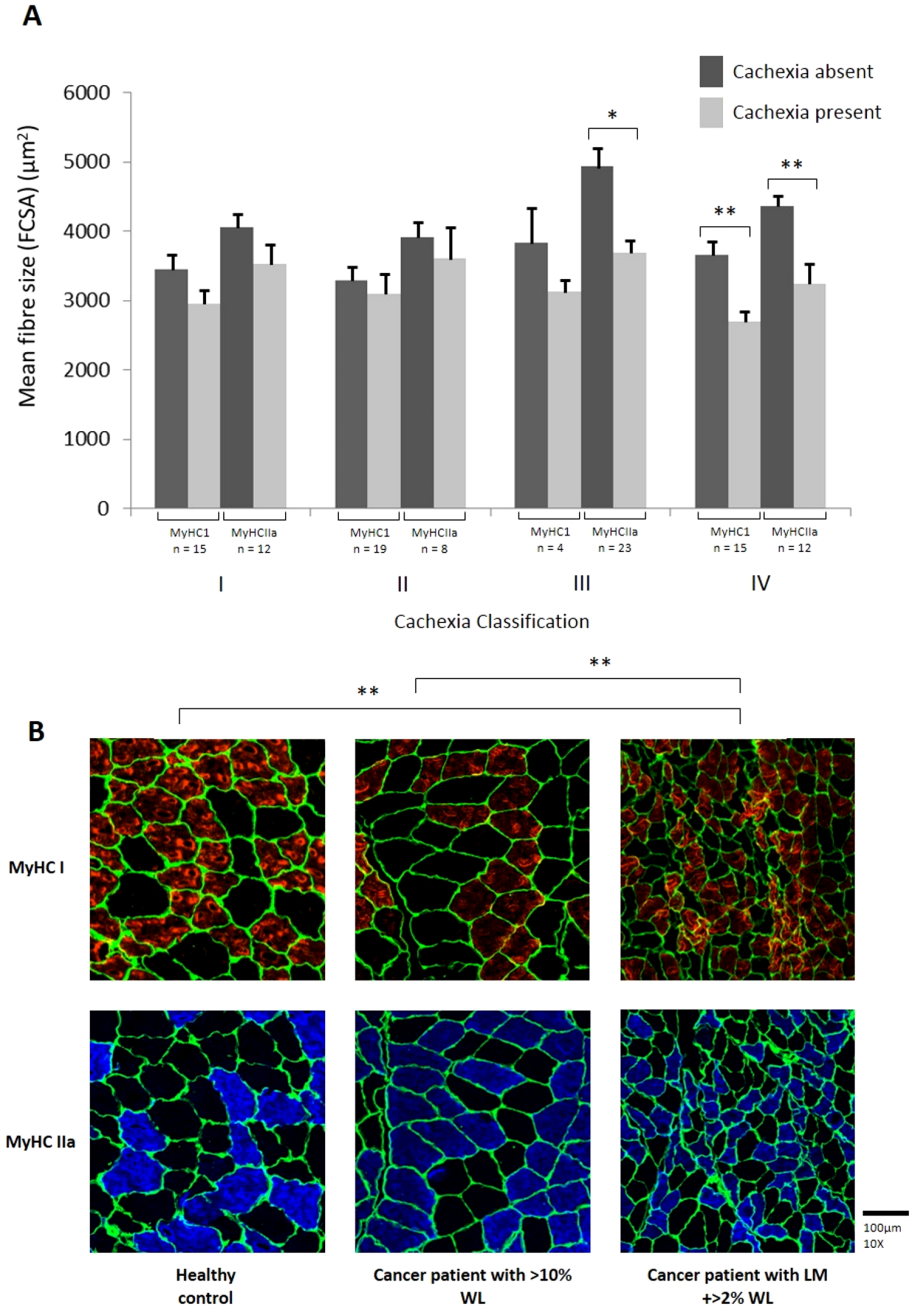
Fibre type cross sectional area (FCSA) according to different definitions of cachexia. (A) Mean (± SEM) fibre size for both MyHC1 and MyHCIIa. A comparison is made between patients with the proposed cachexia definition absent (dark grey) and those with the proposed cachexia definition present (light grey) for the four definitions set out in Methods (I–IV). (*, P<0.05 and **, P<0.01, by Student's t test). (B) Immunohistological sections of muscle for a healthy control, patient with weight loss alone (10.1%) (Group II), and patient with low muscularity and >2% weight loss (Group IV). Laminin is shown in green, MyHC1 shown in red, and MyHCIIa is shown in blue.

If patients were classified as cachectic by LM or LM + >2%WL, fibre size was reduced significantly (all types of myosin heavy chain fibre) when compared with non-cachectic patients and controls (Figure 1A). The association of cachexia with reduced fibre size was not observed if patients were classified according to WL alone. Representative immunohistological sections demonstrating differences in fibre diameter between a healthy control and an individual in Group II versus Group IV is shown in Figure 1B. Immunohistology for type I and IIa resulted in complementary staining in general, whereas fibre type IIb resulted in very low staining intensity as reported elsewhere [19]; therefore quantitative analysis was done only with type I and IIa but not with type IIb (Table 2). As would be expected from a decrease in fibre size, there was a trend across all groups for fibre density to increase in those with cachexia. However, due to large variability, this was not statistically significant. There was no evidence of selective fibre atrophy across any of the classification groups (Table 2).

**Table 2:** Myosin heavy chain fibre data of the patients involved in the study.

	All Patients (n = 27)	Weight Stable (n = 15)	Weight Loss >5% (n = 12)	p- Value	Weight Stable (n = 19)	Weight Loss >10% (n = 8)	p- Value	Normal Muscularity (n = 4)	Low Muscularity (n = 23)	p- Value	Not Low Muscularity + >2% W/L (n = 15)	Low Muscularity + >2% W/L (n = 12)	p- Value
Age	66±2	67±2	64±2	0.321	66±2	65±2	0.791	63±3	66±2	0.370	65±2	66±2	0.882
Sex (M∶F)	24∶3	15∶0	9∶3		18∶1	6∶2		4∶0	20∶3		15∶0	9∶3	
Pre Illness Weight (kg)	85±4	83±5	88±5	0.537	83±4	89±6	0.482	104±12	82±3	**0.018** *	86±5	84±5	0.737
Weight Loss (%)	5±1	0±1	12±1	**0.000** *	2±1	14±2	**0.000** *	5±2	5±2	0.947	1±1	11±2	**0.000** *
BMI at time of biopsy	26±1	27±2	25±1	0.381	29±1	25±1	0.454	33±3	25±1	**0.003** *	28±1	24±1	**0.035** *
Body Fat (kg)	25±1	18±2	18±1	0.980	18±1	17±1	0.848	22±3	17±1	0.103	19±2	16±1	0.255
Free Fat Mass (FFM) (kg)	48±2	49±2	46±2	0.217	48±2	46±2	0.459	59±3	46±1	**0.000** *	51±2	44±2	**0.012** *
Free Fat Mass Index (FFM) (kg/m^2^)	27±0	16±1	15±1	0.303	16±1	15±1	0.511	19±1	15±0	**0.000** *	17±1	14±0	**0.005** *
Mean fibre size (FCSA) (µm^2^)													
MyHC All Data	3588±172	3800±190	3324±297	0.167	3639±180	3468±412	0.514	4509±382	3408±172	**0.020** *	4045±169	2979±234	**0.001** *
Group MyHCI	3232±169	3446±241	2963±225	0.183	3287±206	3101±319	0.621	3832±616	3127±167	0.145	3653±229	2705±159	**0.004** *
Group MyHCIIa	3831±195	4076±195	3524±361	0.167	3924±201	3611±478	0.482	4934±290	3698±193	**0.016** *	4365±164	3238±297	**0.002** *
Total fibre number per mm^2^													
MyHC All Data	1228±147	1007±224	1504±187	0.152	1018±184	1726±252	0.119	937±179	1296±169	0.398	1226±227	1263±183	0.905
Group MyHCI	627±96	525±160	756±92	0.427	506±127	915±133	0.217	406±86	666±112	0.353	653±160	596±97	0.777
Group MyHCIIa	621±94	503±147	769±101	0.067	519±120	863±131	0.163	594±117	693±106	0.706	682±146	676±142	0.976
Fibre Type (%)													
MyHCI	48±3	49±4	41±3	0.128	48±3	38±5	0.079	43±3	46±3	0.691	48±4	42±4	0.230
MyHCIIa	55±2	52±3	59±2	0.107	54±3	58±3	0.388	64±3	53±2	0.099	55±3	55±3	0.978

Data (except gender split) are presented as mean (SEM).

= cachexia group significantly different from the non-cachexia group, (p<0.05 by Student's t test).

Abbreviations – BMI = Body Mass Index, FCSA = Fibre Cross Sectional Area.

### Protein Content

**Figure 2:**
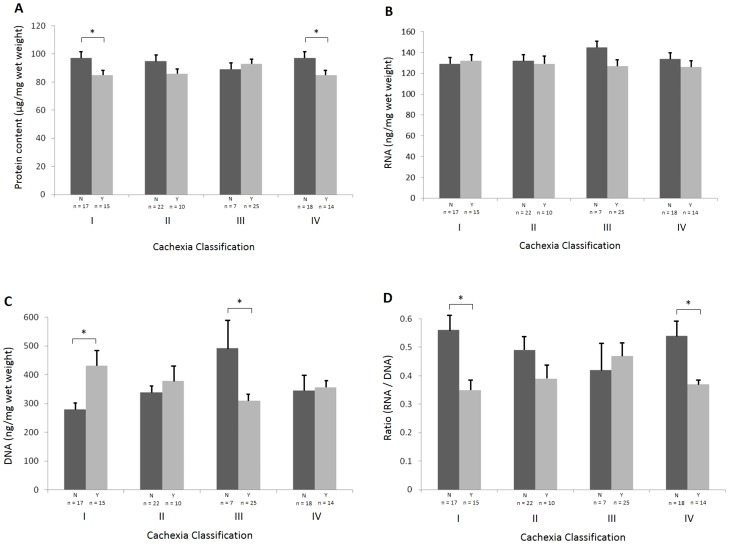
Variations in protein and nucleic acid content according to the different definitions of cancer cachexia. A comparison is made between patients with the proposed cachexia definition absent (dark grey) and those with the proposed cachexia definition present (light grey) for the four definitions set out in the methods (I–IV). (A) Mean (± SEM) wet weight protein content. (B) Mean (± SEM) RNA content. (C) Mean (± SEM) DNA content. (C) Mean (± SEM) RNA/DNA ratio. (*, P<0.05 by Student's t test).

The results for skeletal muscle protein content are shown in Figure 2(A). When compared with non-cachectic patients, muscle protein content was reduced significantly (approximately 13%) in patients with either >5% WL or LM + >2%WL (Figure 2A and table 3). However if the LM criteria were applied alone no difference in the protein content was observed. In addition, patients with >10% WL showed a 10% reduction in protein content when compared with non-cachectic patients but this difference did not reach statistical significance (Figure 2A and table 3).

**Table 3:** Protein, DNA, and RNA content of the patients involved in the study.

	All Patients (n = 32)	Weight Stable (n = 17)	Weight Loss >5% (n = 15)	p- Value	Weight Stable (n = 22)	Weight Loss >10% (n = 10)	p- Value	Normal Muscularity(n = 7)	Low Muscularity (n = 25)	p- Value	Not Low Muscularity + >2% W/L (n = 18)	Low Muscularity + >2% W/L (n = 14)	p- Value
RNA (ng/mg wet weight)	131±6	129±8	132±9	0.859	132±7	129±13	0.847	145±9	127±7	0.220	134±8	126±10	0.520
DNA (ng/mg wet weight)	351±34	279±33	431±54	**0.019** *	338±38	378±66	0.582	493±103	310±28	**0.020** *	345±53	357±34	0.866
Ratio (RNA/DNA)	0.46±0.05	0.56±0.07	0.35±0.04	**0.013** *	0.49±0.06	0.39±0.05	0.254	0.42±0.11	0.47±0.05	0.622	0.54±0.07	0.37±0.02	**0.050** *
Protein content (µg/mg wet weight)	92±2.7	97±4	85±2	**0.015** *	95±4	86±3	0.132	89±4	93±3	0.575	97±4	85±2	**0.035** *
Protein content/RNA	0.77±0.05	0.83±0.09	0.71±0.07	0.273	0.79±0.07	0.75±0.09	0.742	0.63±0.05	0.82±0.07	0.154	0.78±0.08	0.76±0.07	0.827

Data (except gender split) are presented as mean (SEM).

= cachexia group significantly different from the non-cachexia group, (p<0.05 by Student's t test).

### RNA, DNA, and RNA/DNA Ratio

The results for skeletal muscle DNA and RNA content are also shown in Figure 2(B,C, and D) and table 3. RNA content was not significantly different in cachectic patients when compared with non-cachectic patients according to any of the diagnostic criteria (Figure 2B and table 3). In contrast, DNA content was increased by 50% with >5% WL but decreased by ∼40% in patients with LM (Figure 2C). The ratio of RNA/DNA was decreased (approximately 30%) in patients with >5% WL and LM + >2%WL (Figure 2D).

### Autophagy Pathways

**Figure 3:**
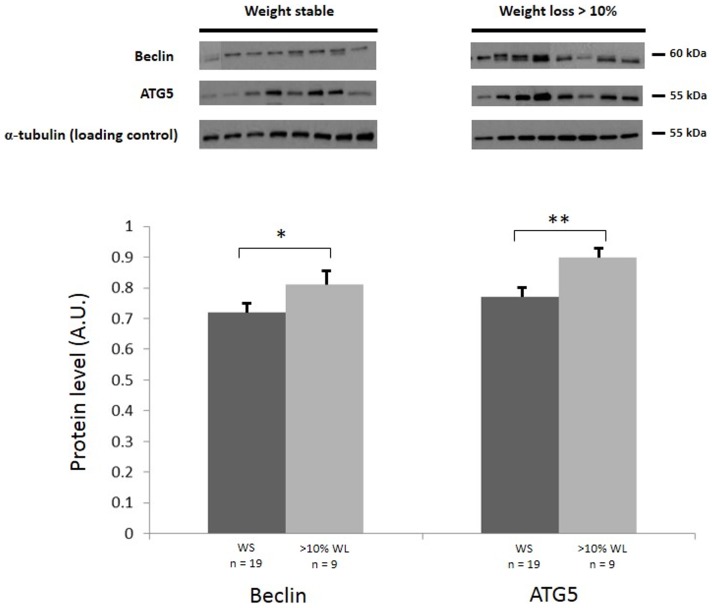
Skeletal muscle Beclin and ATG5 protein levels in patients with or without >10% weight loss (Group II). Western blot analysis with indicated antibodies, α-tubulin was used as a loading control. Graph shows the mean (± SEM) protein level represented in arbitrary units (A.U). (*, P<0.05 and **, P<0.01, by Student's t test).

In patients with >10% WL, Beclin and ATG5 protein levels were increased significantly in cachectic patients when compared with non-cachectic patients (Figure 3). ATG7 and 12 levels were not different in cachectic patients when compared with non – cachectic patients according to any of the diagnostic criteria (Table 3).

### SMAD Signalling

**Figure 4:**
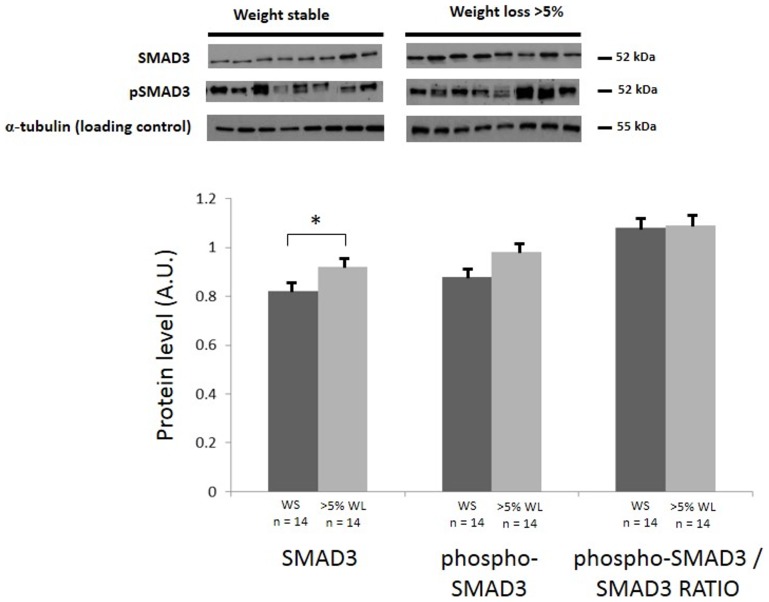
Total SMAD3, phospho-SMAD3 and ratio of phospho-SMAD3/SMAD3 in patients with or without >5% WL (Group I) levels. Western blot analysis with indicated antibodies, α-tubulin was used as a loading control. Graph shows the mean (± SEM) protein level represented in arbitrary units (A.U). (*, P<0.05 by Student's t test).

In patients with >5% WL, SMAD3 protein levels were significantly increased when compared with non-cachectic patients (Figure 4). There were no significant differences in phospho-SMAD3/SMAD3 across any of the groups (Figure 4).

### Inflammatory Pathways

**Figure 5:**
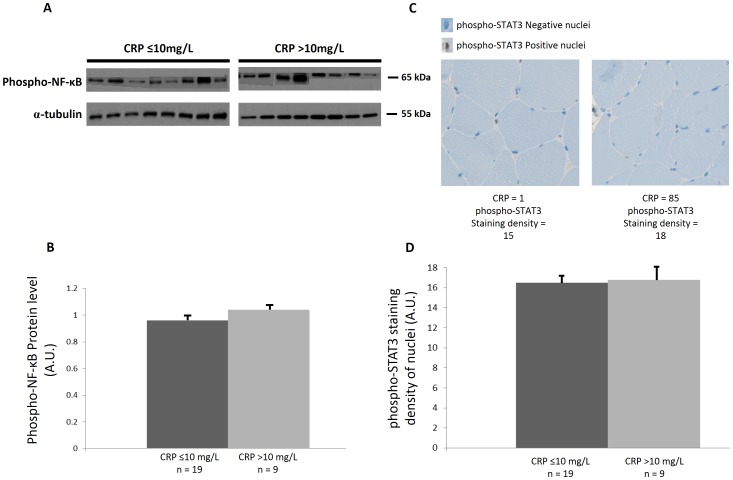
Inflammatory pathways in patients with (CRP >10 mg/L) and without (CRP ≤10 mg/L) systemic inflammation. (A) Western blot analysis in the presence or absence of systemic inflammation with indicated antibodies, α-tubulin was used as a loading control. (B) Graph shows the mean (± SEM) protein level of phospho-NF-κB, represented in arbitrary units (A.U). (C) Representative immunohistochemistry and nuclei count of phospho-STAT3 (area shown is representative of field) of a patient with or without systemic inflammation. (D) Graph shows the staining density of phospho-STAT3 nuclei (A.U.) (± SEM) in the presence or absence of systemic inflammation.

Systemic inflammation was estimated using patients' serum CRP levels (Table 1). Patients were classified as having systemic inflammation if their CRP was ≥10 mg/L. There was no difference in the proportion of patients with or without systemic inflammation according to the definition of cachexia. Levels of phospho-NFκB and phospho-STAT3 were not significantly different in patients with or without cachexia (using any of the definitions: table 4) or with or without systemic inflammation (Figure 5).

**Table 4:** Western blots for protein markers of cellular signalling.

	Weight Stable (n = 14)	Weight Loss >5% (n = 14)	p-Value	Weight Stable (n = 19)	Weight Loss >10% (n = 9)	p- Value	Normal Muscularity (n = 8)	Low Muscularity (n = 20)	p-Value	Not Low Muscularity + >2% W/L (n = 17)	Low Muscularity + >2% W/L (n = 11)	p- Value
Beclin (A.U)	0.73±0.03	0.77±0.04	0.452	0.72±0.02	0.81±0.05	**0.050** *	0.70±0.03	0.77±0.03	0.232	0.72±0.03	0.79±0.04	0.153
ATG5 (A.U)	0.80±0.03	0.83±0.04	0.614	0.77±0.03	0.9±0.04	**0.013** *	0.75±0.05	0.84±0.03	0.137	0.81±0.03	0.82±0.05	0.865
ATG12 (A.U)	0.86±0.04	0.87±0.04	0.898	0.84±0.03	0.93±0.05	0.090	0.83±0.05	0.89±0.03	0.313	0.86±0.03	0.88±0.04	0.720
ATG7 (A.U)	0.89±0.05	0.87±0.05	0.741	0.85±0.04	0.96±0.07	0.136	0.85±0.08	0.89±0.04	0.617	0.89±0.04	0.86±0.06	0.682
phospho-SMAD3 (A.U)	0.88±0.04	0.98±0.04	0.116	0.91±0.04	0.97±0.04	0.343	0.87±0.07	0.95±0.03	0.171	0.90±0.04	0.98±0.04	0.191
SMAD3 (A.U)	0.82±0.03	0.92±0.03	**0.022** *	0.86±0.02	0.89±0.04	0.497	0.88±0.03	0.87±0.03	0.816	0.85±0.02	0.89±0.04	0.348
phospho-SMAD3/SMAD3 (A.U)	1.08±0.06	1.08±0.05	0.972	1.07±0.05	1.11±0.07	0.630	0.98±0.05	1.12±0.05	0.107	1.06±0.05	1.12±0.06	0.467
phospho-NF-κB (A.U)	0.97±0.03	1.00±0.04	0.608	0.96±0.02	1.03±0.05	0.116	0.94±0.03	1.00±0.03	0.258	0.97±0.02	1.01±0.04	0.347
phospho-STAT3 staining density of nuclei (A.U.)	17.9±1.34	15.5±0.62	0.105	17.4±1.03	15.1±0.74	0.170	16.3±0.95	16.7±1.00	0.817	17.2±1.10	15.6±0.80	0.311

Data are presented as mean (SEM). Immunohistology data is shown for phospho-STAT3 and is recorded as staining density of nuclei (A.U.).

= cachexia group significantly different from the non-cachexia group, (p<0.05 by Student's t test).

Abbreviations – A.U = arbitrary units.

## Discussion

### Fibre Size

The diagnostic criterion for cancer cachexia has long been based on weight loss alone [1] and can reflect loss in either fat or lean tissue compartments. Given that the key tissue loss in cancer cachexia is considered to be skeletal muscle, a recent consensus process suggested that the diagnostic criteria for cachexia should also take account of low baseline levels of muscularity [1]. In the present study, when patients were classified as cachectic or not according to ≥5% weight loss there was no significant difference in whole body muscularity (FFMI) or muscle fibre CSA. In contrast, when patients were classified according to low muscularity and ≥2% weight loss, FFMI was decreased and fibre cross sectional area was also significantly reduced (Figure 1). Such findings demonstrate that heterogeneity in relation to low muscularity and fibre atrophy may be reduced according to the clinical definition of cachexia. This finding may be important especially when considering inclusion criteria for clinical trials that aim to test the efficacy of drugs targeted at reversal of muscle wasting in cancer patients. The reduction in fibre size in all MyHC isoforms observed in the present study is consistent with previous animal [20] and human studies of cancer cachexia [4]–[7]. The rectus muscle of patients with oesophago-gastric cancer cachexia has been shown to lose all type MyHC content as well as undergo a reduction in fibre size [4]. Equally, in pancreatic cancer patients with cachexia, both type I and type II MyHC protein levels were decreased by 45% when compared with controls [6].

### Fibre Type

In order to study differences in muscle fibre morphology and composition within the different cachexia categories, we performed immunohistochemical analysis of human muscle samples. For that, we first established and validated the staining methods for the myosin heavy chain antibodies specific for the different fibre types (I, IIa, and IIb). Staining for type I and IIa fibres resulted in strong specific staining specificity, however only weak staining was observed against type IIb MyHC, this finding has also been reported elsewhere [19]. The predominant types of MyHC fibre in rectus abdominis muscle are I and IIa and only <8% of type IIb positive fibres have previously been described [21]. Of the adult skeletal isoforms, each are expressed to varying degrees in both mouse and human skeletal muscle. However, although MyHCIIb is highly expressed at both the messenger RNA (mRNA) and protein level in murine skeletal muscle, evidence to date suggests that this isoform is effectively only expressed at the mRNA level in a very small subset of specialised muscles in the adult human [22]. As mentioned above, MyHCIIb expression is typically associated with high forces of contraction combined with rapid contractile characteristics and it has been suggested that the contractile characteristics of MyHCIIb may be incompatible with the biomechanical constraints of larger muscles [23], which may account for the lack of specificity found in the rectus muscle of our patient population.

In cachexia there is conflicting evidence as to whether there is selective loss of fibre type. There was no evidence for selective loss of fibre type in the present study (Table 2). Evidence from animal models suggests that Type II fibres are targeted selectively [24], with relative preservation of type I fibres in fasting [25], exposure to glucocorticoids [26], sepsis [27] and in the gastrocnemius muscle of the C26 model of cancer cachexia [28], [29]. Models of cardiac cachexia, however have suggested a trend to selective loss of type I fibres and an increase in type II fibre [30]. Furthermore, not all groups have demonstrated Type I and II fibre differences even in animals. Indeed in a recent study of the C26 cachectic mouse model, both glycolytic and oxidative fibres of (extensor digitorum longus) EDL muscle underwent wasting [20], whilst in a previous study using the same mouse model there was a significant increase in the amount of MyHCIIb and a significant decrease in the amount of type 1 MyHC in soleus muscle [31]. It is currently not entirely clear which type of fibres are affected in human cancer cachexia, however, in patients with oesophago-gastric cancer cachexia early loss of all MyHC isoforms has been reported [4].

The activity patterns of a muscle are also key in determining phenotype. If muscle cells are recruited infrequently they develop into fast/glycolytic units whereas if they are recruited more often, they form slow/oxidative units. In the C26 mouse model of cancer cachexia, there have been reports of switching of myosin isoforms in the soleus muscle of cachectic mice [31]. In pancreatic cancer patients with cachexia, no difference in the ratio of fast/slow myosin isoform was demonstrated compared with controls [6].

### Muscle RNA, DNA, and Protein Content

In the present study, when compared with non-cachectic patients, muscle protein content was reduced significantly (approximately 13%) if patients were classified as cachectic by either >5% WL (p = 0.015) or LM + >2%WL (p = 0.035), and by 10% in patients with >10% WL. Protein content expressed in relation to wet weight of muscle has been shown to decrease progressively (in excess of 50%) in the gastrocnemius muscle of mice bearing the MAC-16 tumour [32]. This suggests that not only is there loss of fibre diameter, but that the quality of the fibre is altered with loss of either sarcoplasmic or myofibrillar protein. Such changes in fibre composition may contribute to the reduced muscle mechanical quality (force per unit cross-sectional area) observed in human cancer cachexia [33].

A reduction in both RNA content and activity in skeletal muscle has been attributed to a depression of protein synthesis in mice bearing the MAC16 tumour [32] . In the present study, RNA content was unaltered in cachectic patients (classified either with >5% WL or LM + >2% WL) compared with non-cachectic patients. A reduction in the RNA content in the muscle of mice bearing the Ehrlich ascites tumour has also been reported, but this occurred later than the observed depression in the rate of protein synthesis [34]. Whether muscle protein synthesis is depressed in human cancer cachexia remains to be resolved [10].

In a murine model of cancer cachexia DNA content of the gastrocnemius muscle has been shown to remain relatively constant, despite the finding of a decrease in protein and RNA content [32]. The current study demonstrated DNA content was increased by >50% with >5% WL but decreased by 40% in patients with LM (Figure 2C). Because mature myofibre nuclei are thought to be mitotically inactive, increased DNA content in skeletal muscle cells suggests activation of satellite cells [35] or infiltration by other cell types such as inflammatory cells or adipocytes. In the LM group, the decrease in DNA may be due to pre-existing age-related sarcopenia or other causes of muscle atrophy (e.g. immobilisation) and may relate to muscle specific apoptosis and reduction in cell number in keeping with a reduction in muscle mass on CT scanning. The diametrically opposite changes in muscle DNA content dependent on whether patients are classified according to weight-loss or low muscularity again underpin the potential diverse mechanisms whereby older cancer patients may develop a low level of muscularity.

The issue of whether nuclear domain size is reduced in cancer cachexia remains to be resolved. In particular, whether apoptosis in skeletal muscle is increased in cancer cachexia and the degree to which DNA content is maintained or not via a compensatory increase in myonuclear number (possibly via satellite cell nuclei incorporation) is not known. Features of cachexia such as hypogonadism (resulting in low testosterone) or systemic inflammation (associated with high IL-6) could influence such regenerative capacity. In the current study RNA/DNA was altered in the cachectic patients (independent of definition) compared with the non- cachectic patients. This may be due to the interplay of the mechanisms described above.

### Mechanisms

Skeletal muscle atrophy may occur as a result of decreased synthesis, increased degradation or both [36]. In mice bearing the MAC-16 adenocarcinoma, muscle loss is due to the combination of reduced synthesis and increased degradation [37]. Similarly Samuels et al demonstrated reduced protein synthesis and increased degradation in skeletal muscle co-incident with the onset of cachexia in mice implanted with the C26 murine model [38].

#### Degradation Pathways

The majority of signalling pathways contributing to muscle atrophy in pre-clinical models are mediated through activation of the ubiquitin-proteasome proteolytic pathway (UPP) [39]. The muscle-specific E3 ubiquitin ligases, MuRF-1 and MAFbx/atrogin-1 are up regulated in animal models of acute atrophy [40], [41], and MuRF1 selectively targets the myofibrillar protein myosin heavy chain resulting in muscle wasting [8]. However, the role of the E3 ligases in human cachexia is less well defined. In the current study we chose not to measure directly these pathways as results from our previous investigation on a similar cohort of patients found no up regulation using a transcriptomics approach [42], this has also been validated recently in a separate cohort of patients with gastric cancer [43]. In the present study autophagy proteins (ATG) 5, 7, 12, and beclin 1 were studied. These proteins are necessary for autophagy due to their role in autophagosome elongation [44]. When patients were classified according to >10% WL, Beclin and ATG5 protein levels were significantly increased in cachectic patients when compared with non-cachectic patients. In a previous study in a similar cohort of patients, the autophagy related genes GABRAPL1 and BNIP3 were increased in rectus muscle biopsies from cachectic versus non-cachectic patients [42]. In normal muscle, low-protein diets up-regulate autophagy that leads to the loss of muscle mass at least partially through lysosomal degradation [45]. Intriguingly, under other circumstances decreased autophagy can also lead to muscle atrophy.

#### Systemic Inflammation

Systemic inflammation is thought to be a major mediator of cancer cachexia [10]. However, the relationship between inflammation in the systemic compartment versus muscle and the relationship of either to muscle loss in humans is not clear. In the systemic compartment, Il-6 is thought to be a major mediator and may signal within target organs via STAT-3. Alternatively, both IL-1 and TNF alpha may signal via NF-kB. NF-kB regulation of muscle atrophy is predominantly executed by promoting proteasome-mediated degradation [46]. Activation of NF-kB has been detected in both physiological and pathological atrophic conditions such as denervation, unloading, aging, cancer, sepsis, diabetes, and such atrophy can be reversed by pharmacologic or genetic NF-kB inhibition [47]. In the present study although there was evidence for systemic inflammation in a proportion of patients, no significant difference was found in the levels of phospho-NFkB or phospho-STAT3 across any of the definitions of cachexia or in those with or without evidence of systemic inflammation. It is possible that inflammatory mediators have their main effects on muscle atrophy via central mechanisms mediated via the CNS [48].

#### SMAD3

It has been suggested that binding of myostatin to the ActRIIB receptor results in the phosphorylation of two serine residues of SMAD2 or SMAD3. This leads to the assembly of SMAD2/3 with SMAD4 to the heterodimer that is able to translocate to the nucleus and activate transcription of target genes [49]. One of the known downstream targets of SMAD signalling is MyoD, a transcriptional factor that is involved in skeletal muscle development and takes part in the repair of damaged skeletal muscle [50]. Moreover, SMAD signalling targets other genes such as myf5 and myogenin, known to be important for myogenesis [51]. Myostatin is upregulated in cachexia and in states of muscle paralysis [52]. Myostatin/ActRIIB activates SMAD2/3 signalling and importantly SMAD2/3 inhibition completely desensitises ActRIIB-induced muscle atrophy [13]. Inhibition of myostatin by a dominant negative ActRIIB promotes muscle hypertrophy independent of muscle satellite cell recruitment consistent with a direct signalling effect on muscle catabolism [13]. When patients were classified as cachectic according to >5% WL, SMAD3 protein levels were significantly increased in cachectic patients when compared with non-cachectic patients. Equally there was a similar (but not significant) increase in phospho-SMAD3 associated with >5% weight loss. It is not known whether such increased protein levels indicate increased pathway activity independent of any alteration in the ratio of phospho-SMAD3/SMAD3.

### Limitations of Study

It is important to appreciate that the majority of patients in the present series will have had some degree of age-related sarcopenia, that this will necessarily co-exist with any cancer specific loss of skeletal muscle mass and that the diagnostic criteria used in the present study will not necessarily separate one from the other. The current study was not longitudinal and it was therefore not possible to document active muscle loss. It is also important to recognise that when patients were divided into different diagnostic categories the sample size in individual categories may have limited the ability to detect a statistical difference or not. This was an exploratory study and provides the basis for a larger study with adequate statistical power for definitive analysis.

### Conclusions

In the present study, when the diagnostic criteria for cachexia included both a measure of low muscularity and weight loss, muscle fibre size, protein content and RNA/DNA content were all reduced. Such consistent findings were not observed when cachexia was diagnosed based on weight-loss or low muscularity alone. Whereas fibre type is not targeted selectively, muscle fibre size, biochemical composition and pathway phenotype can vary according to whether the criteria for cachexia include both a measure of low muscularity and weight loss. Such findings suggest that current diagnostic criteria identify groups of patients with different skeletal muscle phenotypes. Identification of a more homogeneous patient cohort for musculo-centric intervention trials may require use of combined criteria.
