# Correction: Clinical Classification of Cancer Cachexia: Phenotypic Correlates in Human Skeletal Muscle

**DOI:** 10.1371/journal.pone.0314953

**Published:** 2024-12-02

**Authors:** Neil Johns, Shinji Hatakeyama, Nathan A. Stephens, Martin Degen, Simone Degen, Wilfried Frieauff, Christian Lambert, James A. Ross, Ronenn Roubenoff, David J. Glass, Carsten Jacobi, Kenneth C. H. Fearon, the PLOS ONE Editors

Concerns were raised about potential splice lines and about similarities in data shown across blot panels in Figs 3–5 of this article [1].

The authors noted that errors were made when assembling these figures. They provided images of the original blots (S1 File) for these figures and explained that lanes from the original blots were rearranged during figure preparation to cluster samples by experimental group.

S1 File shows aligned experimental and control data for each patient: each lane in S1 File represents data for the same patient across all blots. S1 Table with this notice shows how the original lane (patient) numbers in S1 File correspond to results shown in the published figures. As is indicated in S1 Table and S1 File, corresponding lanes of the blots shown together in each figure panel do not show data for the same patients, and the α-tubulin blot in each figure panel includes data from multiple experimental groups.

S2 File includes the raw quantification data derived from the western blots and from which the graphs in Figs 3–5 were generated. The authors confirmed that in quantifying the results of the western blot analyses, each individual’s experimental data was normalized to their corresponding control blot (α-tubulin) data, as is shown in S2 File. For pSMAD analyses, the pSMAD and SMAD3 densitometry values were each normalized to α-tubulin and then the ratio was calculated. In Fig 5A and the corresponding densitometry analysis, phospho-NF-κB values were normalized to α-tubulin. The authors also included total NF-κB blots in the experiment (S3 File) but no major regulation was observed across samples and the total NF-κB data were not included in the quantitative analysis.

In Table 2 of the published article [1], p values were erroneously reported as 0.000 in several cases. These should instead indicate “p<0.001” in each case.

In Fig 5D of the published article [1], the numbers on the x axis should be CRP<10 n = 15, and CRP>10 n = 6.

Additional raw data for this article [1] are provided in S4–S10 Files.

The *PLOS ONE* Editors consider that the data in S1 File suffice to address the splicing and similarity concerns raised about the published figures. However, a member of *PLOS ONE*’s Editorial Board advised that given the way this study’s data are presented it is difficult to ascertain whether the original data support the results reported in the article.

The corresponding author, Kenneth C.H. Fearon, passed away in 2016. Any further questions about this work can be directed to Neil Johns.

## Supporting information

S1 Table S1 File lane numbers for which western blot data are shown in the published Figs 3–5 [1]. Fig 5A shows the lower bands from the phospho-NF-κB blots (p-NFKB) in S1 File. Figure labels do not correctly represent the results shown in α-tubulin panels; see S1 File for details. For Beclin, ATG5, SMAD3, and pSMAD3, symbols indicate differences in blot labels comparing Fig 3 and Fig 4 and S1 File: labels in S1 File are * WL 5%; ** no weight loss; ^+^ healthy control; ^&^ WL 10%.
(DOCX)

S1 File Original western blot panels for all samples. (JPG)

S2 File Densitometry analysis of western blot data used to generate tables in Figs 3–5. (XLSX)

S3 File Total NF-κB western blot data. (DOCX)

S4 File pSMAD western blot data. (PDF)

S5 File Total SMAD3 western blot data. (PDF)

S6 File pNF-κB western blot data. (DOCX)

S7 File Spreadsheet used to collect and analyze all raw data generated for article [1]. (CSV)

S8 File Supporting data for Fig 5C (PPTX)

S9 File Supporting data for Fig 5C. (PPTX)

S10 File Supporting data for Fig 5C. (PPTX)
