# Supplementary material for: Correction: Clinical Classification of Cancer Cachexia: Phenotypic Correlates in Human Skeletal Muscle
Source: PLoS One. 2024 Dec 2;19(12):e0314953. doi: 10.1371/journal.pone.0314953 (PMC11611210; doi:10.1371/journal.pone.0314953)
Supplement: S1 Table — Fig 5A shows the lower bands from the phospho-NF-κB blots (p-NFKB) in S1 File. Figure labels do not correctly represent the results shown in α-tubulin panels; see S1 File for details. For Beclin, ATG5, SMAD3, and pSMAD3, symbols indicate differences in blot labels comparing Fig 3 and Fig 4 and S1 File: labels in S1 File are * WL 5%; ** no weight loss; + healthy control; & WL 10%. (DOCX) [file pone.0314953.s001.docx]

|  | Figure lane number: | | 1 | 2 | 3 | 4 | 5 | 6 | 7 | 8 | 1 | 2 | 3 | 4 | 5 | 6 | 7 | 8 |
| --- | --- | --- | --- | --- | --- | --- | --- | --- | --- | --- | --- | --- | --- | --- | --- | --- | --- | --- |
| Figure panel description & S1 File lane numbers | Figure panel label: | | Weight stable | | | | | | | | Weight loss >10% | | | | | | | |
|  | Figure 3 | Beclin | 31 | 15* | 16 | 17* | 18 | 19 | 20 | 21 | 3 | 7 | 13 | 14 | 24 | 25 | 28** | 29 |
|  |  | ATG5 | 15* | 16 | 17* | 18 | 19 | 20 | 21 | 26* | 3 | 7 | 13 | 14 | 24 | 25 | 29 | 30 |
|  |  | α-tubulin | 14 | 15 | 16 | 17 | 18 | 19 | 20 | 21 | 4 | 5 | 6 | 7 | 8 | 9 | 10 | 11 |
|  | Figure panel label: | | Weight stable | | | | | | | | Weight loss >5% | | | | | | | |
|  | Figure 4 | SMAD3 | 4 | 6 | 19 | 20 | 8 | 9 | 10^+^ | 11 | 2 | 3^&^ | 17 | 23 | 13^&^ | 14^&^ | 15 | 24^&^ |
|  |  | pSMAD3 | 8 | 9 | 18 | 19 | 20 | 21 | 11 | 31 | 23 | 24^&^ | 25^&^ | 26 | 27^&^ | 13^&^ | 14^&^ | 15 |
|  |  | α-tubulin | 1 | 2 | 3 | 4 | 5 | 6 | 7 | 8 | 12 | 13 | 14 | 15 | 16 | 17 | 18 | 19 |
|  | Figure panel label: | | CRP ≤ 10mg/L | | | | | | | | CRP > 10mg/L | | | | | | | |
|  | Figure 5 | p-NF-κB | 2 | 3 | 4 | 15 | 16 | 17 | 18 | 19 | 10 | 11 | 12 | 13 | 24 | 25 | 29 | 30 |
|  |  | α-tubulin | 14 | 15 | 16 | 17 | 18 | 19 | 20 | 21 | 4 | 5 | 6 | 7 | 8 | 9 | 10 | 11 |

**S1 Table.** S1 File lane numbers for which western blot data are shown in the published Figures 3, 4, and 5 [1]. Figure 5A shows the lower bands from the phospho-NF-κB blots (p-NFKB) in S1 File. Figure labels do not correctly represent the results shown in α-tubulin panels; see S1 File for details. For Beclin, ATG5, SMAD3, and pSMAD3, symbols indicate differences in blot labels comparing Figure 3 and Figure 4 and S1 File: labels in S1 File are * WL 5%; ** no weight loss; ^+^ healthy control; ^&^ WL 10%.
