# Supplementary figures and images for: Correction: Clinical Classification of Cancer Cachexia: Phenotypic Correlates in Human Skeletal Muscle
Source: PLoS One. 2024 Dec 2;19(12):e0314953. doi: 10.1371/journal.pone.0314953 (PMC11611210; doi:10.1371/journal.pone.0314953)

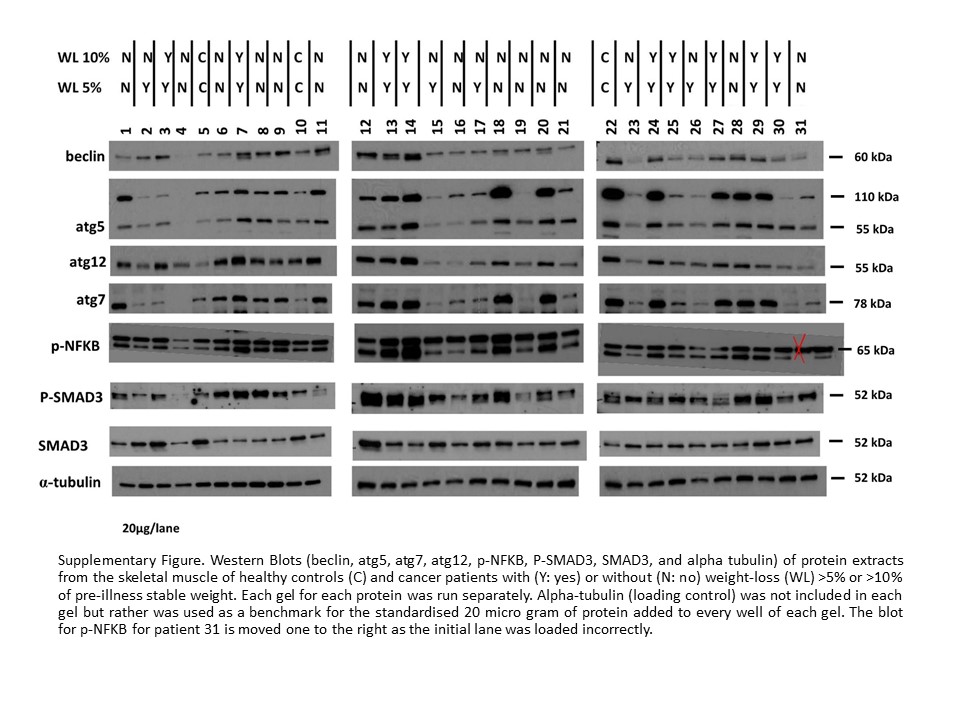

Supplement: S1 File — (JPG) [file pone.0314953.s002.jpg]

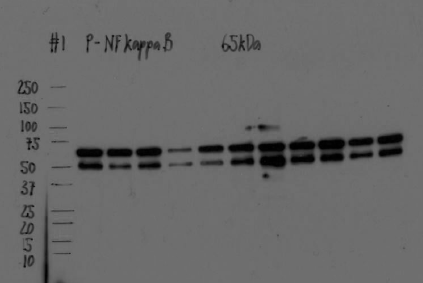


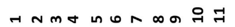


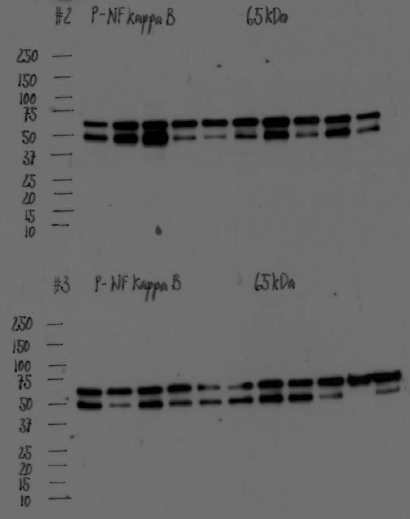


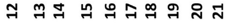

Supplement: S6 File — (DOCX) [file pone.0314953.s007.docx]

## Slide 1
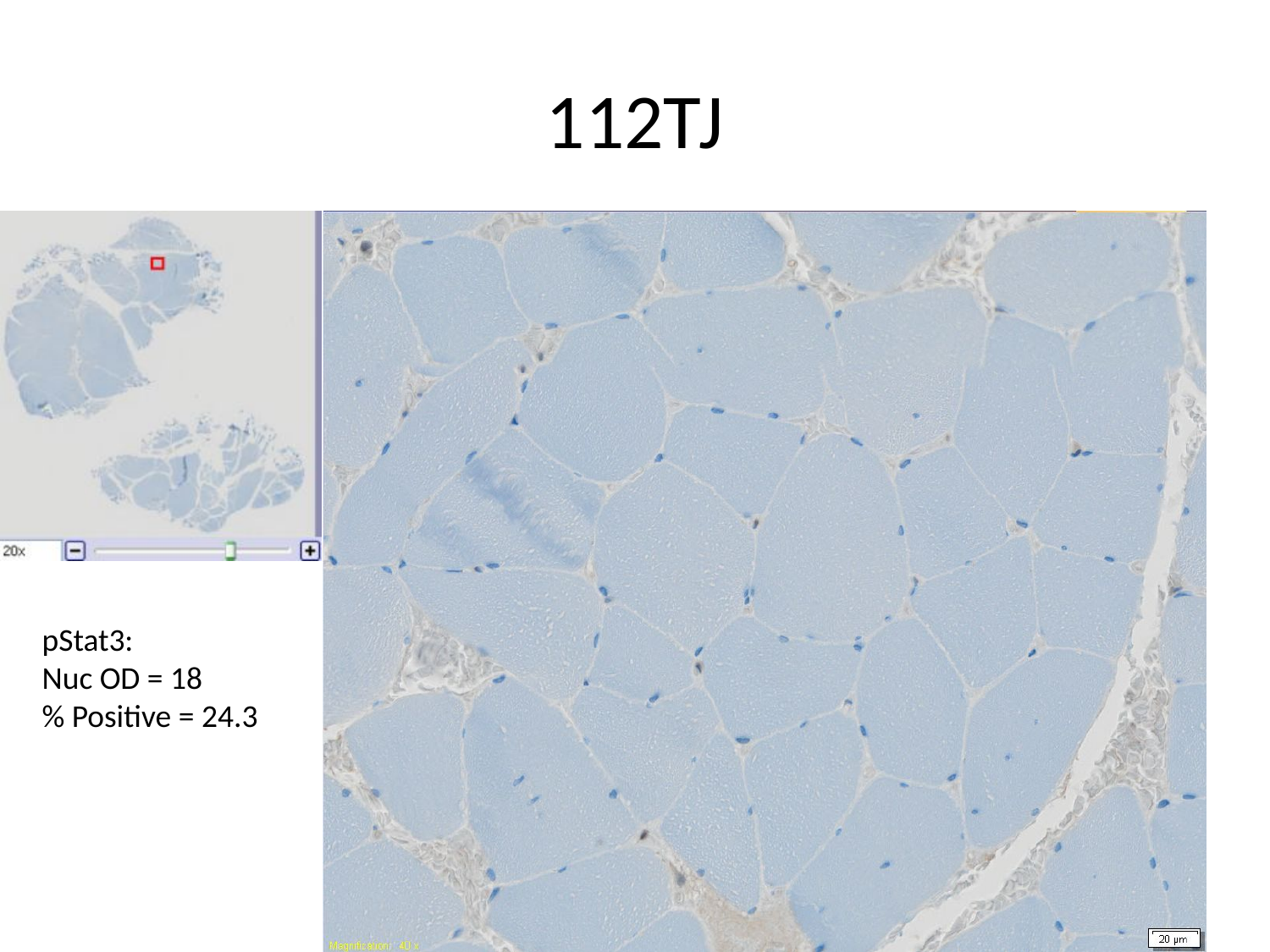

# 112TJ
pStat3:
Nuc OD = 18
% Positive = 24.3

## Slide 2
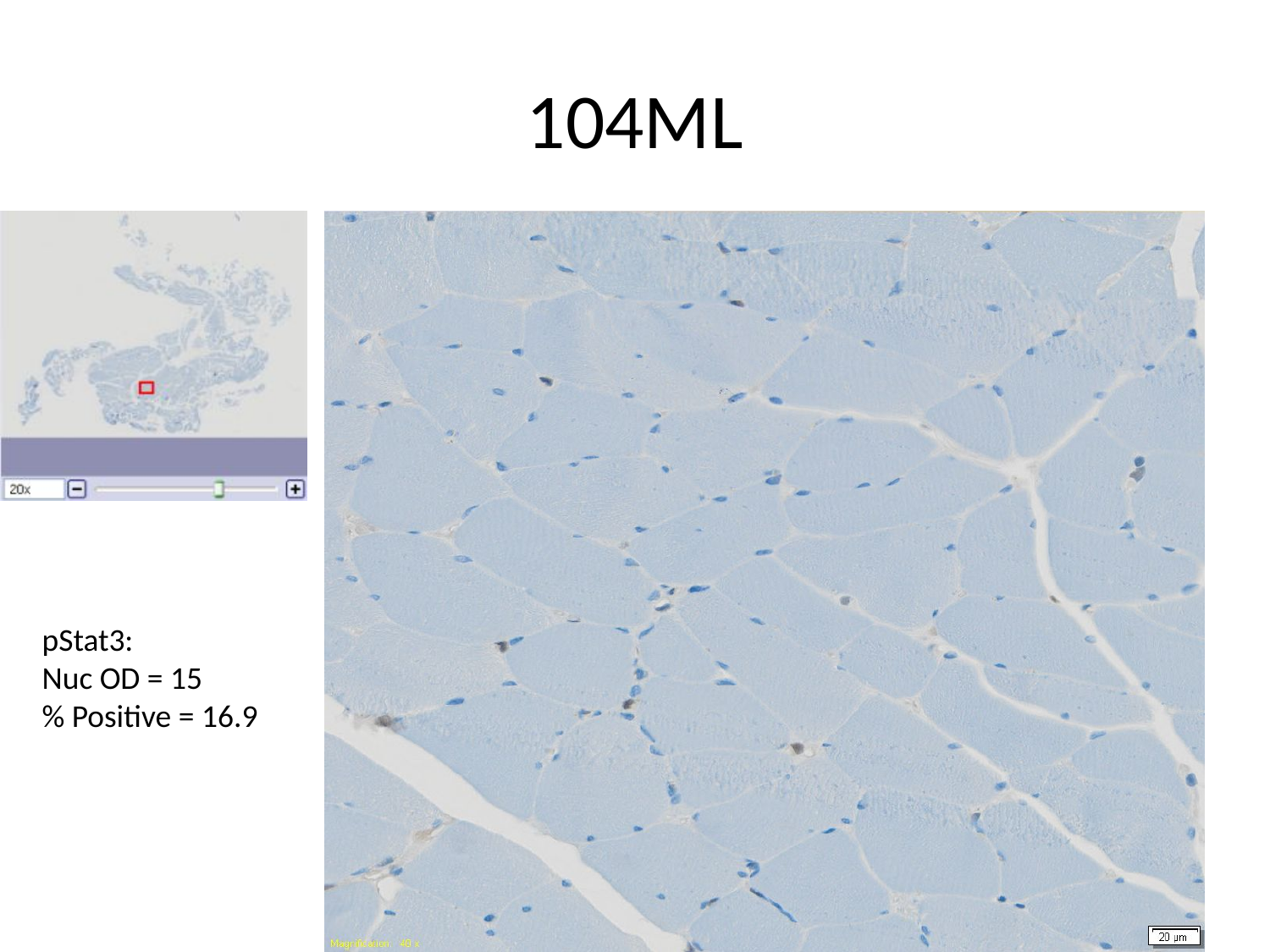

# 104ML
pStat3:
Nuc OD = 15
% Positive = 16.9

## Slide 3
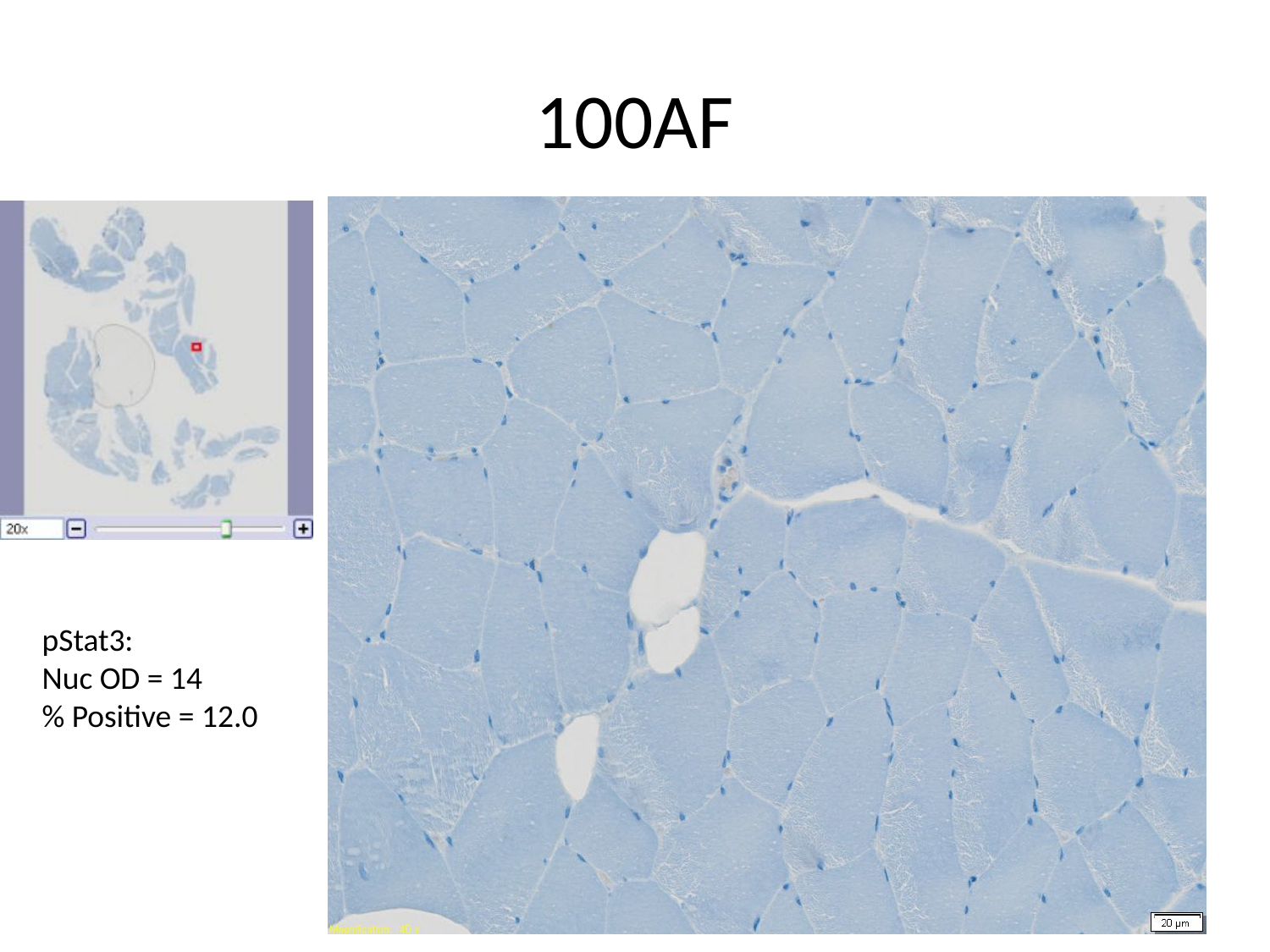

# 100AF
pStat3:
Nuc OD = 14
% Positive = 12.0

Supplement: S9 File — (PPTX) [file pone.0314953.s010.pptx]
