# Supplementary material for: Correction: Clinical Classification of Cancer Cachexia: Phenotypic Correlates in Human Skeletal Muscle
Source: PLoS One. 2024 Dec 2;19(12):e0314953. doi: 10.1371/journal.pone.0314953 (PMC11611210; doi:10.1371/journal.pone.0314953)
Supplement: S3 File — (DOCX) [file pone.0314953.s004.docx]

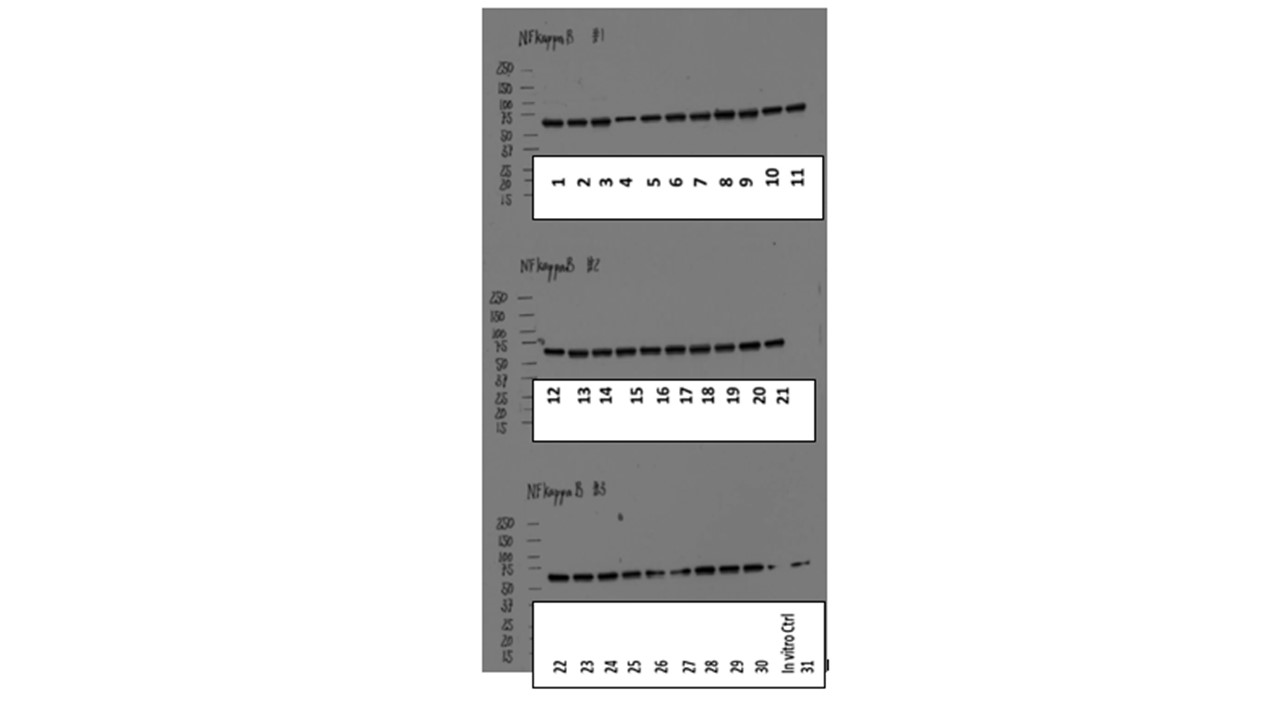


All samples show the correct band for NFkappaB at 65kDa, but the control band is weak and not well exposed
