# Supplementary material for: Correction: Clinical Classification of Cancer Cachexia: Phenotypic Correlates in Human Skeletal Muscle
Source: PLoS One. 2024 Dec 2;19(12):e0314953. doi: 10.1371/journal.pone.0314953 (PMC11611210; doi:10.1371/journal.pone.0314953)
Supplement: S4 File — (PDF) [file pone.0314953.s005.pdf]

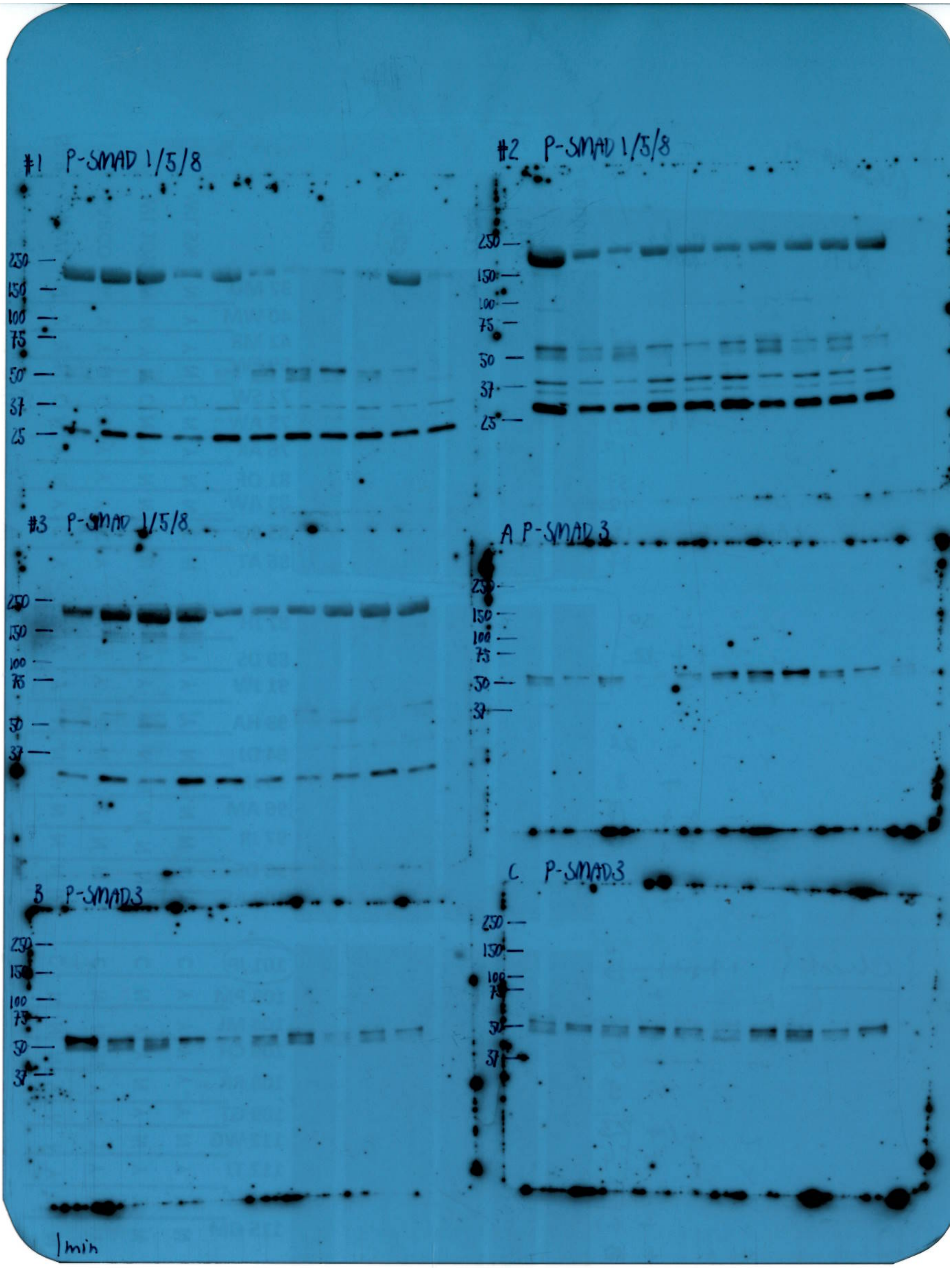
#1 P-SMAD 1/5/8
#2 P-SMAD 1/5/8
#3 P-SMAD 1/5/8
A P-SMAD3
B P-SMAD3
C P-SMAD3
1min
