# Supplementary material for: Correction: Clinical Classification of Cancer Cachexia: Phenotypic Correlates in Human Skeletal Muscle
Source: PLoS One. 2024 Dec 2;19(12):e0314953. doi: 10.1371/journal.pone.0314953 (PMC11611210; doi:10.1371/journal.pone.0314953)
Supplement: S5 File — (PDF) [file pone.0314953.s006.pdf]

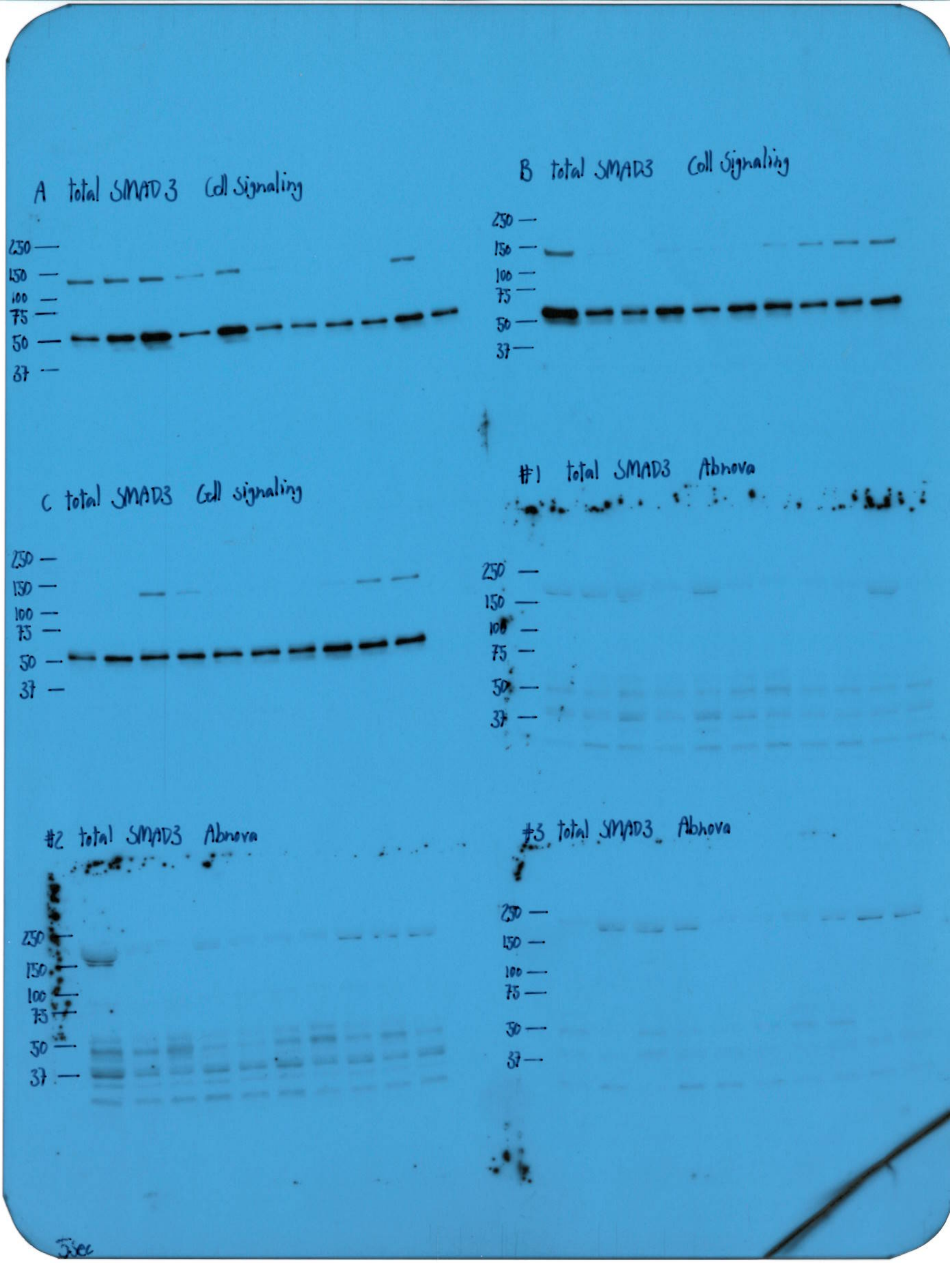
A total SMAD 3 Cell Signaling
250
150
100
75
50
37
B total SMAD3 Cell Signaling
250
150
100
75
50
37
C total SMAD3 Cell signaling
250
150
100
75
50
37
#1 total SMAD3 Abnova
250
150
100
75
50
37
#2 total SMAD3 Abnova
250
150
100
75
50
37
#3 total SMAD3 Abnova
250
150
100
75
50
37
5sec
