# Supplementary material for: Correction: Clinical Classification of Cancer Cachexia: Phenotypic Correlates in Human Skeletal Muscle
Source: PLoS One. 2024 Dec 2;19(12):e0314953. doi: 10.1371/journal.pone.0314953 (PMC11611210; doi:10.1371/journal.pone.0314953)
Supplement: S8 File — (PPTX) [file pone.0314953.s009.pptx]

## Slide 1
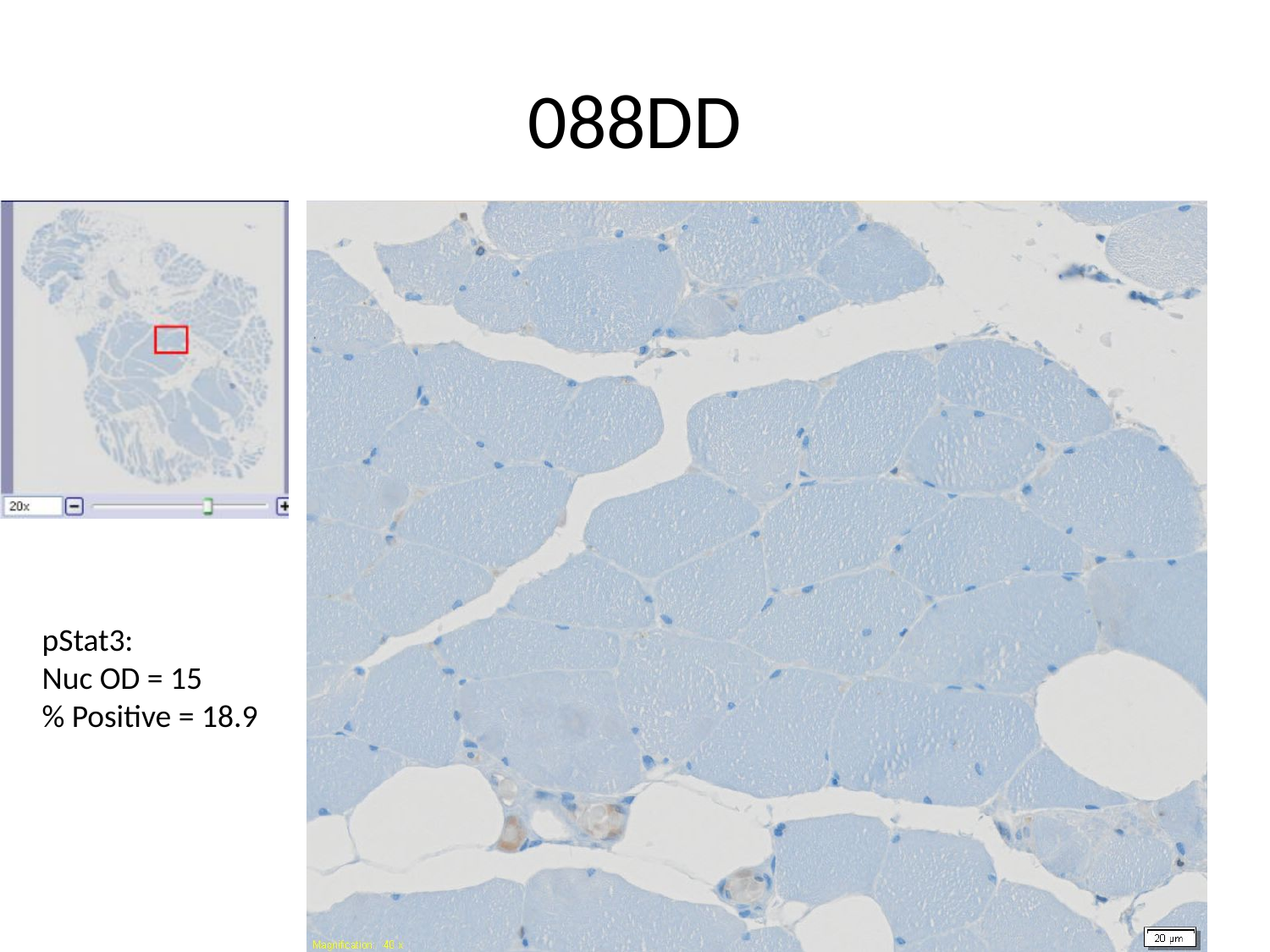

# 088DD
pStat3:
Nuc OD = 15
% Positive = 18.9

## Slide 2
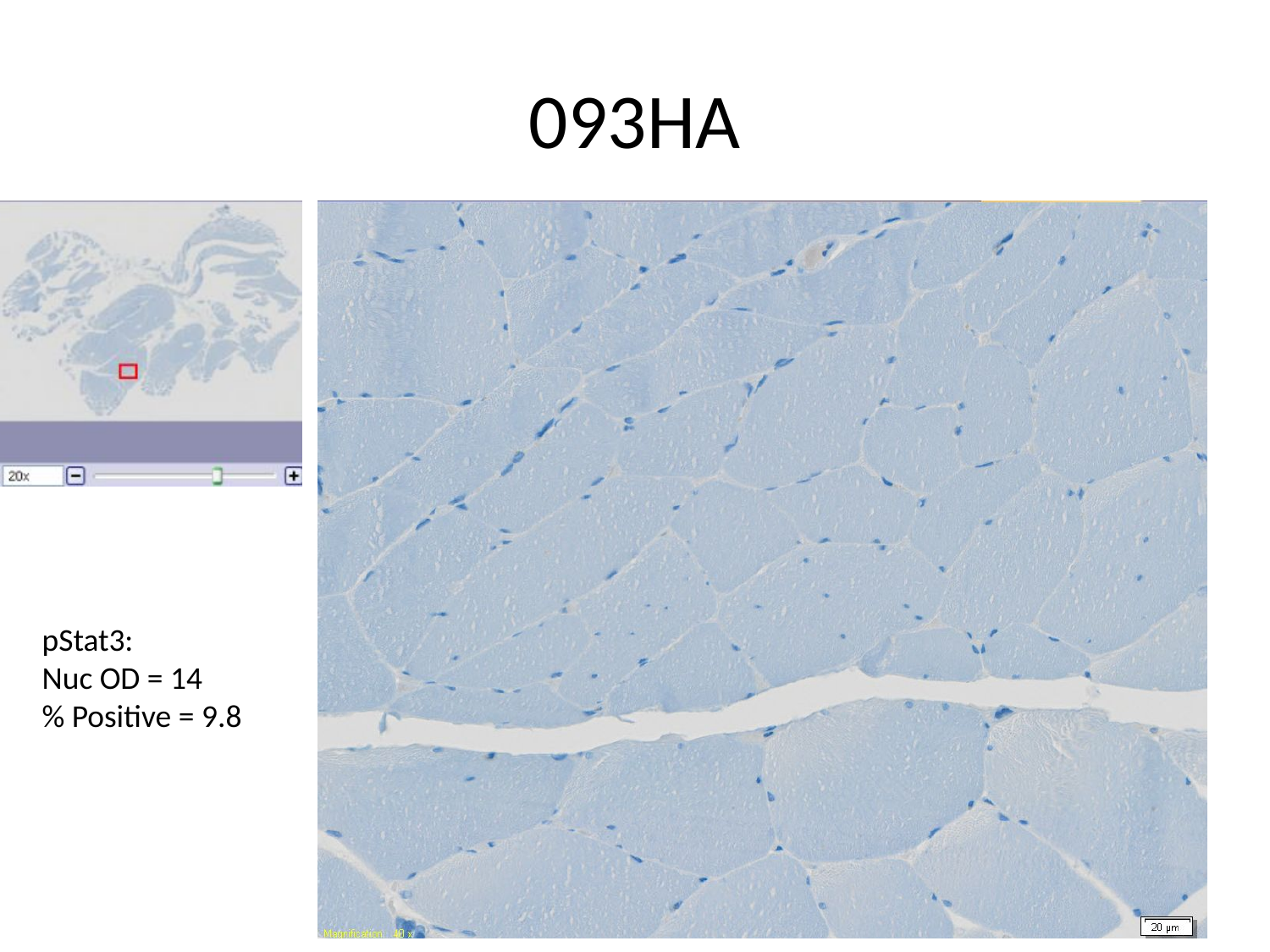

# 093HA
pStat3:
Nuc OD = 14
% Positive = 9.8

## Slide 3
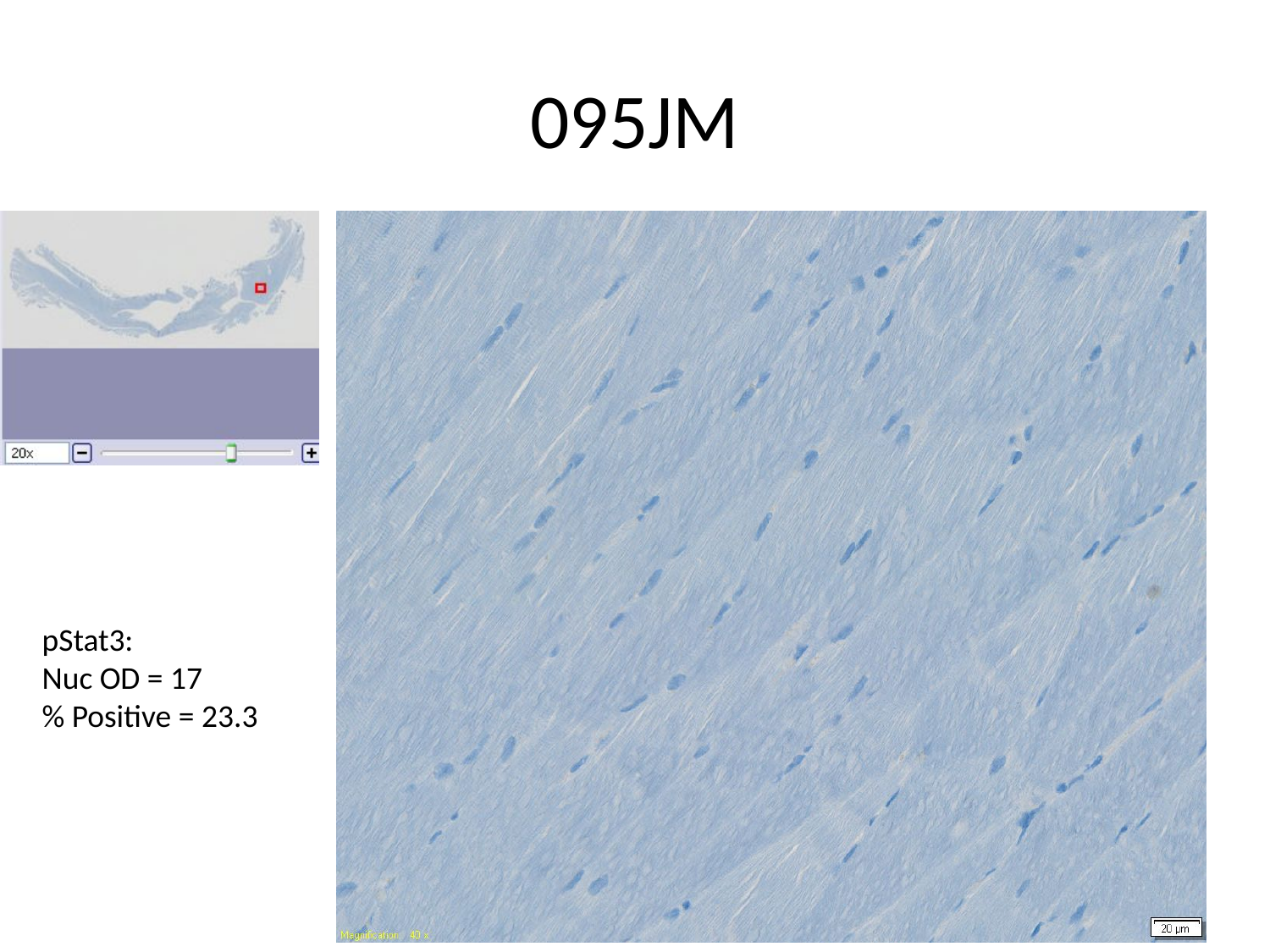

# 095JM
pStat3:
Nuc OD = 17
% Positive = 23.3

## Slide 4
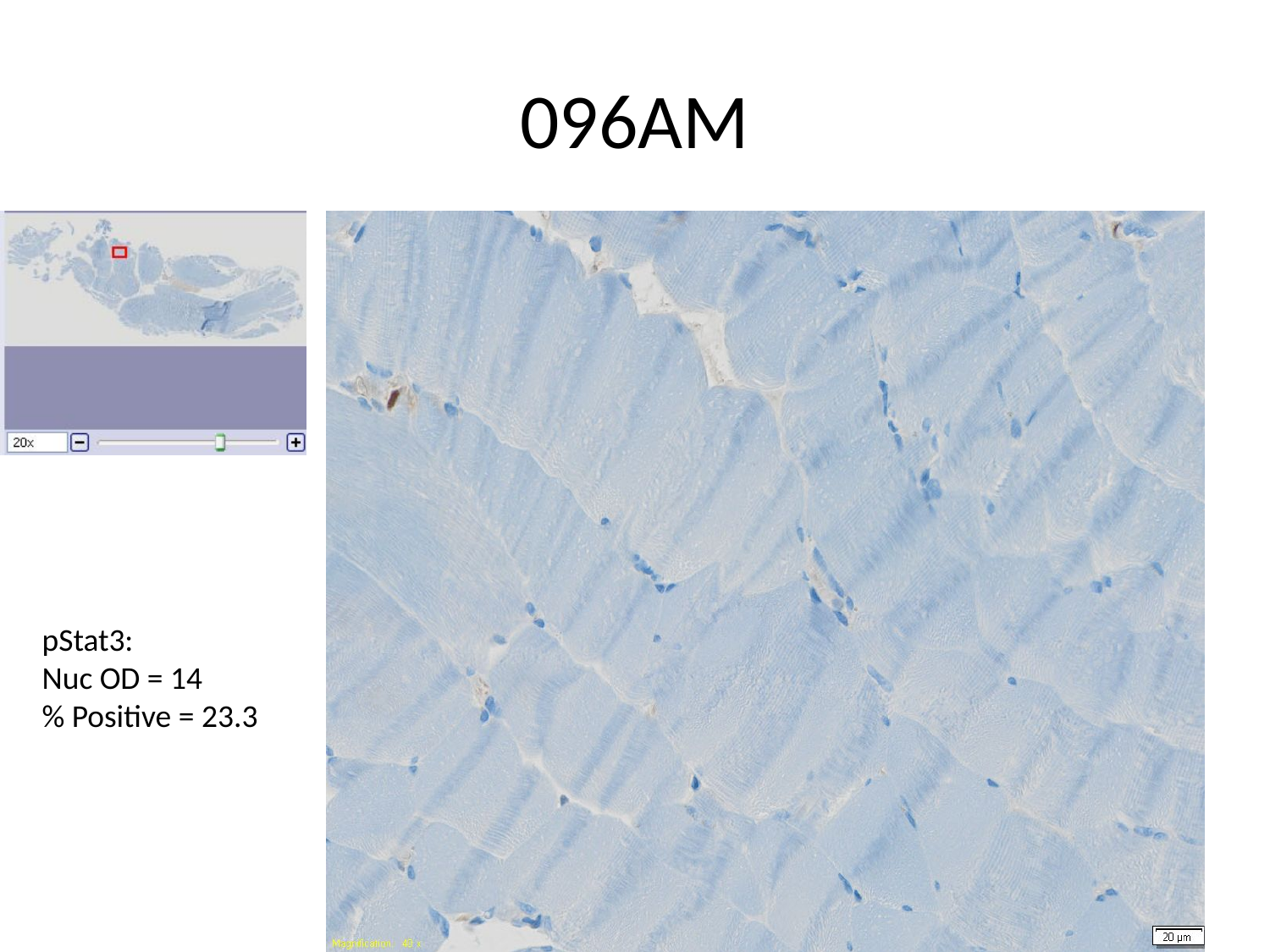

# 096AM
pStat3:
Nuc OD = 14
% Positive = 23.3
