# Supplementary material for: Correction: Clinical Classification of Cancer Cachexia: Phenotypic Correlates in Human Skeletal Muscle
Source: PLoS One. 2024 Dec 2;19(12):e0314953. doi: 10.1371/journal.pone.0314953 (PMC11611210; doi:10.1371/journal.pone.0314953)
Supplement: S10 File — (PPTX) [file pone.0314953.s011.pptx]

## Slide 1
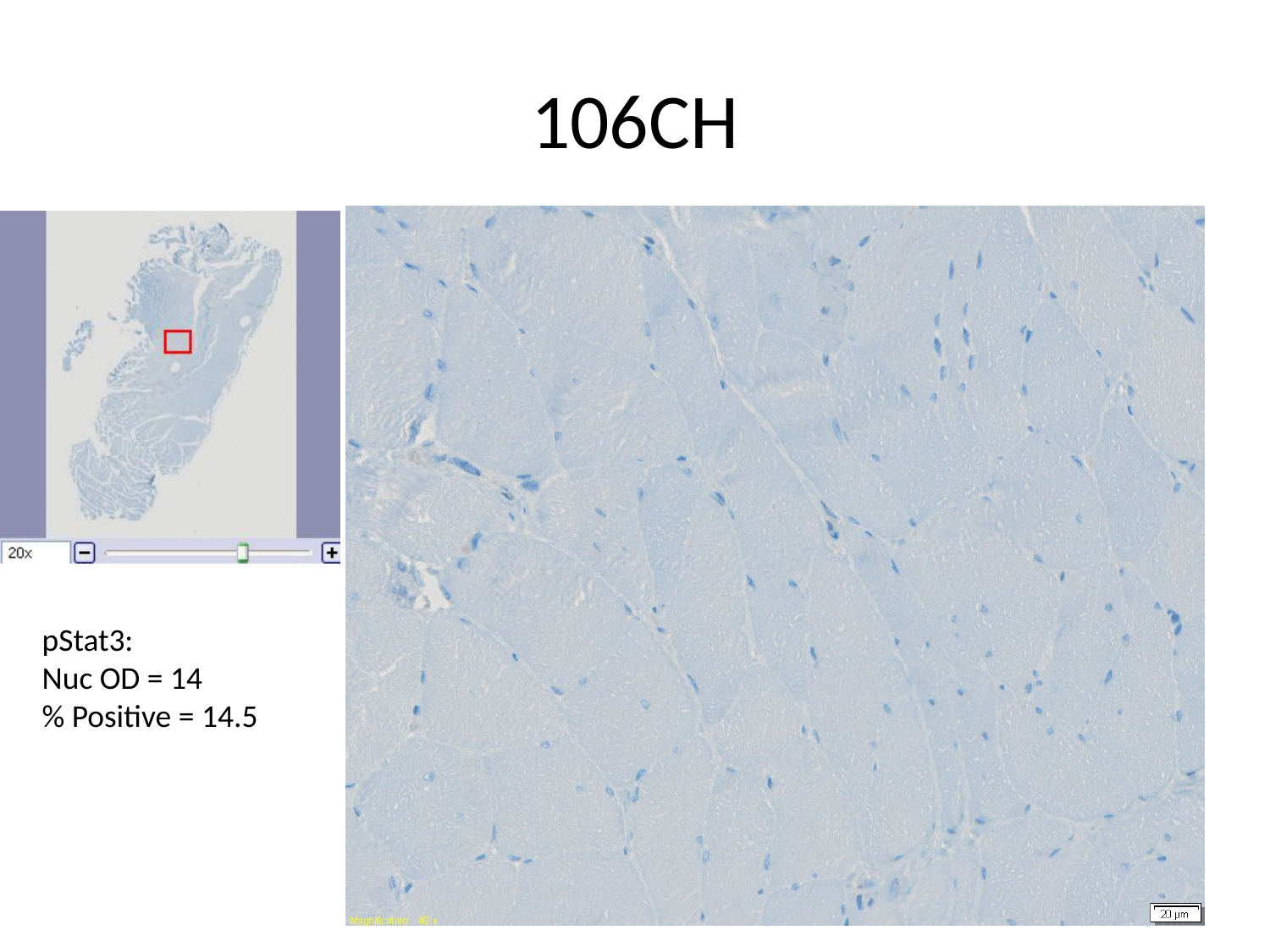

# 106CH
pStat3:
Nuc OD = 14
% Positive = 14.5

## Slide 2
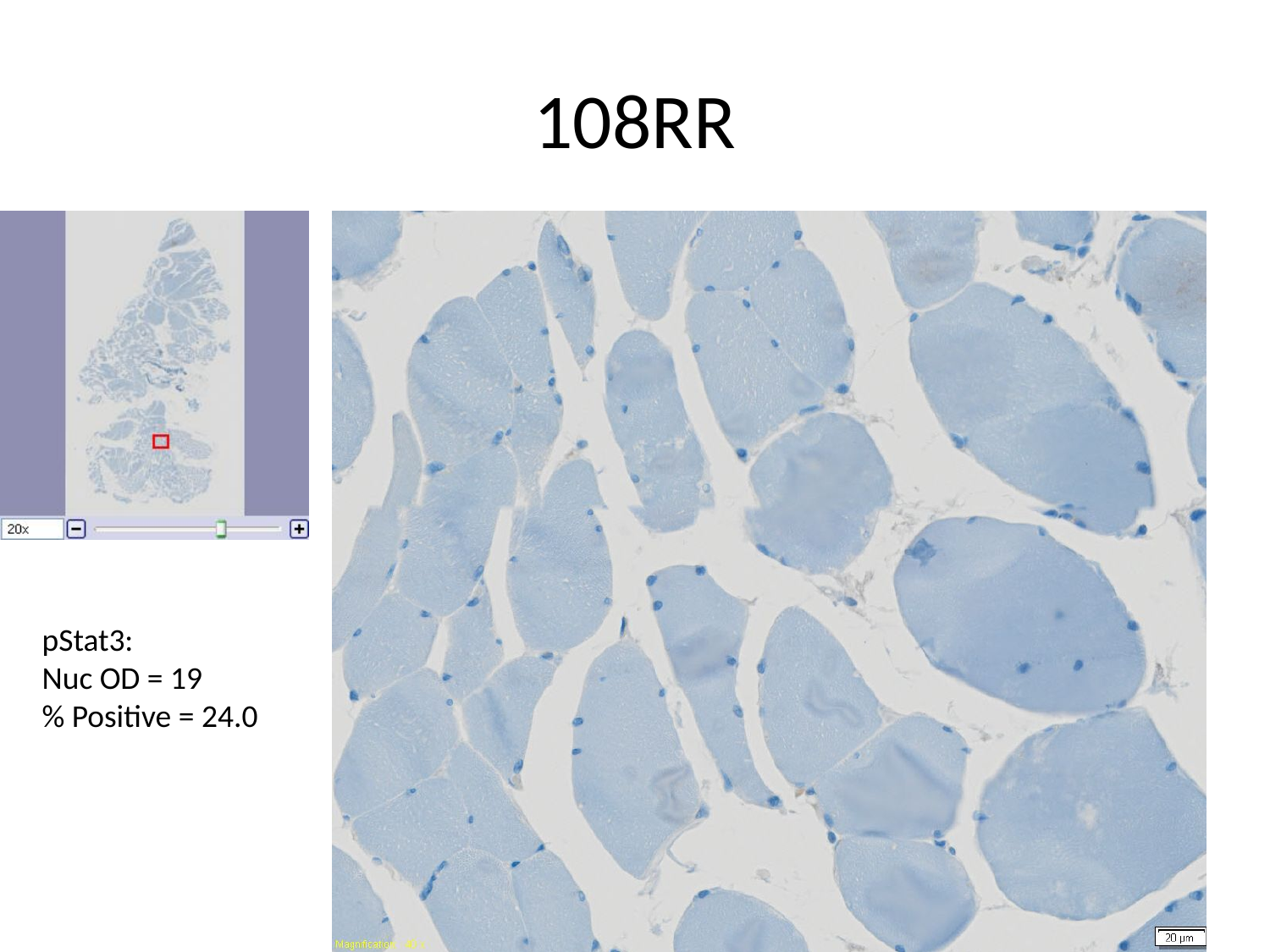

# 108RR
pStat3:
Nuc OD = 19
% Positive = 24.0

## Slide 3
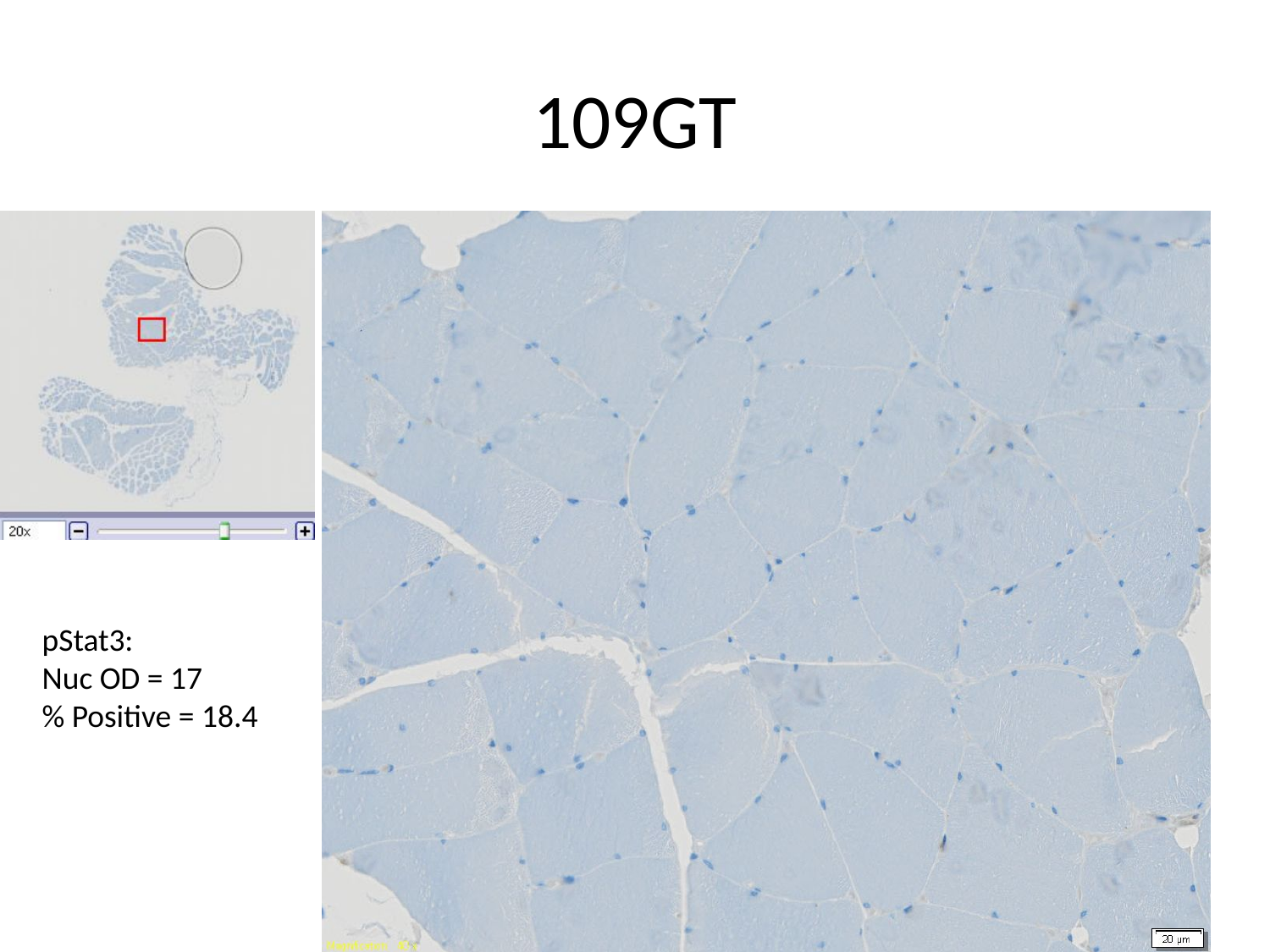

# 109GT
pStat3:
Nuc OD = 17
% Positive = 18.4

## Slide 4
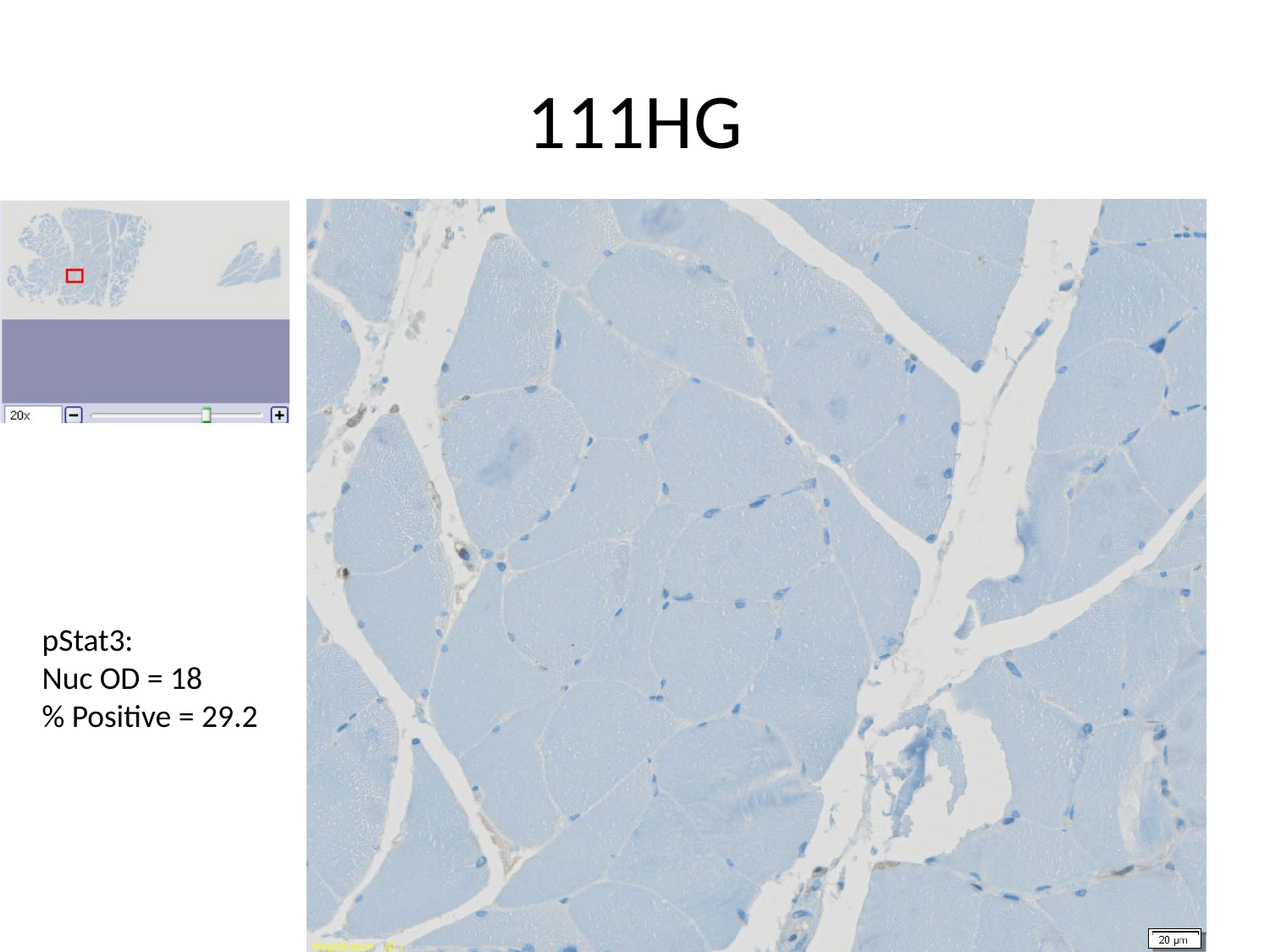

# 111HG
pStat3:
Nuc OD = 18
% Positive = 29.2
